# Excess-Methane CO_2_ Reforming over Reduced KIT-6-Ni-Y Mesoporous Silicas Monitored by In Situ XAS–XRD

**DOI:** 10.1021/acs.energyfuels.3c02994

**Published:** 2023-11-17

**Authors:** Katarzyna Świrk Da Costa, Paulina Summa, Jithin Gopakumar, Youri van Valen, Patrick Da Costa, Magnus Rønning

**Affiliations:** † Department of Chemical Engineering, Norwegian University of Science and Technology (NTNU), 7491 Trondheim, Norway; ‡ Institut Jean Le Rond d’Alembert, Sorbonne Univeristé, CNRS UMR 7190, 78210 Saint-Cyr-l’Ecole, France; § Faculty of Energy and Fuels, AGH University of Science and Technology, 30-059 Cracow, Poland

## Abstract

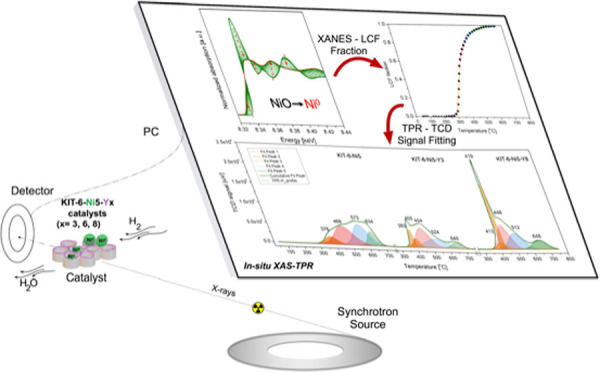

Making Europe less dependent on imported fuels requires a long-term strategy. Low-quality natural gas and biogas could be used to mitigate the energy crisis, and excess-methane dry reforming has the potential to upgrade a mixture of CH_4_ and CO_2_. Herein, nickel-based KIT-6-supported catalysts (KIT-6-Ni) were modified with 3, 6, and 8 wt % of yttrium (Y/Ni molar ratio of 0.5, 1.07, and 1.5) to investigate the influence of this element on catalytic performance. Yttrium was well dispersed, preserving the mesopore structure of KIT-6. The yttrium addition increased the total basicity, contributing to a lower deactivation factor and remarkably stable syngas production compared to the catalyst containing only Ni. In situ XAS-XRD showed that Y allowed for the reduction of Ni^2+^ to Ni^0^ at significantly lower temperatures. A significant difference in the rate of reduction was observed for the studied samples. The analysis showed that the data of linear combination fitting of XANES can demonstrate linear fits with the reduction rate of NiO. The reduction rate of bulk and weakly interacting NiO increased for Y-promoted samples, while a decrease in the rate was registered for species strongly interacting with the support. The latter decreased more with increasing yttrium content. EXAFS analysis showed that Ni is completely reduced in the samples. Under excess-methane dry reforming conditions, the studied catalysts remained fully reduced and showed resistance to sintering of Ni particles. HRTEM results of KIT-6-Ni5-Y8 indicated that metallic Ni particles were decorated by Y_2_O_3_ and/or NiYO_3_. The dominant deactivation mechanism was the carbon encapsulation of Ni particles and the growth of filaments.

## 1 Introduction

Considering the European Union’s newly set targets, aiming at a 55% reduction in greenhouse gas (GHG) emissions by 2030 and climate neutrality by 2050, the energy sectors of all 27 EU Member States are determined to take all necessary steps. The aim of *the European Green Deal* is to reduce greenhouse gas emissions in a fair, cost-effective, and competitive manner.^1^ CO_2_ emissions from fuel combustion have rapidly increased, reaching the highest growth since preindustrial levels in 2018, with a total of 33.5 Gt CO_2_. The lockdowns caused by COVID-19 only temporarily reduced the level of CO_2_ emissions, which continued to increase in 2020 and the first half of 2021. The *Global Energy Review* of the International Energy Agency (IEA) provides an annual update on the world’s latest energy and emissions trends.^2^ Global energy-related CO_2_ emissions peaked at 33.4 GtCO_2_ in 2019, followed by an extraordinary decline of 1.9 GtCO_2_ (5.7%) in 2020. In 2021, the CO_2_ concentration has largely bounced back to prepandemic levels, which is associated with an expected 4.6% increase in global energy demand. Considering the recent incidents associated with the natural gas release on the Baltic Sea, an increase in total GHG emissions is likely to be observed in 2022.

To limit the growing CO_2_ emissions, associated with energy production and use, the European Union recognizes the following methods: increasing the efficiency of energy production, carbon capture and utilization processes (CCU), carbon capture and storage processes (CCS), and/or the use of renewable energy sources.^3^ CCU and CCS are key technologies to reach this goal. Although the development of CO_2_ storage technology is of great importance, minimization of CO_2_ production and its use to produce valuable goods are highly preferable. In CCU processes, carbon dioxide can be used as a raw material in the syntheses of desired chemicals, which in turn improves the reputation of CO_2_ by treating it as a valuable feedstock.^8^

Dry reforming of methane (DRM) is one of the processes assuming the direct conversion of CO_2_ in the presence of CH_4_. This endothermic reaction produces a mixture of H_2_ and CO, known as the synthesis gas (eq 1).^4−6^
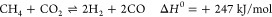
1

Excess-methane dry reforming can be applied in low-quality natural gas and biogas fields containing a considerable amount of CO_2_. In the view of escalating energy crisis in Europe, this seems to be a promising alternative to secure supplies of imported fuel. Purification of the fields can be realized with the aid of DRM, which allows to avoid the separation of CO_2_ from the feed and/or releasing it into the atmosphere. Accordingly, the reaction can use CO_2_ directly and provide lower purification costs for the fields. DRM produces valuable synthesis gas, which is a resource for the manufacture of useful value-added products, e.g., for the synthesis of long-chain hydrocarbons or oxygenate chemicals (acetic acid, dimethyl ether, and oxo-alcohols).^7^

The development of dry reforming catalysts has progressed rapidly over the past decades. Nickel catalysts became one of the most interesting materials for CH_4_ and CO_2_ conversion as methane activation occurs on Ni^0^ and is the rate-limiting step of the reaction.^6,8,9^ The dissociation of CH_4_ requires interaction with metallic Ni capable of releasing C and H_2_, and an oxide support or promoter that can activate CO_2_. In this regard, the nature of the carrier material is critical as it not only affects Ni dispersion but also acts as a catalyst component. Further advantages of using nickel-based materials are their high efficiency, low cost, and abundance.^5^

KIT-6 (Korea Advanced Institute of Science and Technology, KIT) mesoporous silicas have attracted significant attention mostly due to their multifunctional properties. Their enhanced textural features, such as high surface area (ranging 733 m^2^/g, type-IV isotherm), and well-defined 3D interconnected mesopores make them good candidates for being used as supports in the field of catalysis.^10−12^ Mesoporous silica-supported catalysts are commonly studied for DRM, but unlike the widely reported SBA-15 materials, KIT-6 catalysts are starting to become more popular and the number of reports is increasing over the years.^5,11,13−19^ Among these studies, KIT-6-Ni catalysts used in DRM were either unpromoted^11,12,15,16,19^ or promoted with different metals (La,^14,18^ Ce,^17^ and Ru^17^). To the best of our knowledge, only one report (our previous study) can be found on DRM over yttrium-promoted KIT-6-Ni catalysts.^13^ The results from the characterization of yttrium-promoted materials showed higher reducibility of NiO, a larger size of Ni crystallites after reduction and DRM tests, and better nickel dispersion in the channels of the KIT-6 carrier compared to Y-free catalysts. In addition, larger Ni particles were observed on the outer surface of the support, which may be related to the catalytic selectivity toward the carbon formation reaction. After DRM, Ni^0^ and Y_2_O_3_ were recorded. The presence of the Y_2_Si_2_O_7_ phase cannot be ruled out due to the similar *d* spacing as the Y_2_O_3_ phase. Moreover, there was no indication of Ni–Y alloy formation.

In the present study, we focused on Ni-based KIT-6 catalysts impregnated with different loadings of yttrium (3, 6, and 8 wt %) and the fixed amount of nickel to examine catalytic performance in excess-methane dry reforming. Compared to our previous study, we intended to test a series of catalysts with an increased Y/Ni molar ratio of 0.5, 1.07, and 1.5 for KIT-6-Ni5-Y3, KIT-6-Ni5-Y6, and KIT-6-Ni5-Y8, respectively. Moreover, for the first time, KIT-6-Ni-Y catalysts were tested with a higher content of methane in the reaction feed (53 vol %). Furthermore, in this work, in-depth examination is presented by synchrotron radiation techniques (XAS-XRD). This allows us to understand the course of reduction of nickel oxide (4% H_2_/He 750 °C for 90 min) and the nature of the nickel active phase under CH_4_/CO_2_ reforming conditions (700 °C, 1 bar, and excess of methane). In the current work, we attempt to estimate reduction rates based on the data points originating from linear combination fitting (LCF) of Ni XANES spectra.

## 2 Experimental Part

### 2.1 Catalyst Preparation

#### 2.1.1 Synthesis of the KIT-6 Support

4 g of Pluronic P123 triblock copolymer (Sigma-Aldrich) was dissolved in 7.47 g of aqueous solution of HCl (37%) (ACS reagent, MERCK) and the temperature of the solution was increased to 35 °C. The mixture was vigorously stirred for 6 h and then 4 g of *n*-butanol (Sigma-Aldrich) was added dropwise. After 1 h, 8.6 g of tetraethyl orthosilicate (TEOS) (Sigma-Aldrich) was introduced while stirring at 35 °C. The mixture was left to react for the next 24 h.^20^ Thereafter, the solution was transferred to a Teflon bottle for hydrothermal synthesis. The synthesis was carried out under static conditions at 100 °C for 24 h. The obtained product was filtered and dried at 70 °C overnight. To remove the template, calcination was performed at 600 °C for 6 h (heating rate of 2.5 °C/min).^13^

#### 2.1.2 Preparation of KIT-6-Ni and KIT-6-Ni-Y Catalysts

Wet coimpregnation was used to prepare the KIT-6-supported Ni–Y catalysts. An aqueous solution of 80 mL with Ni(NO_3_)_2_·6H_2_O (ACS reagent, MERCK) (KIT-6-Ni5), or Ni(NO_3_)_2_·6H_2_O and Y(NO_3_)_3_·6H_2_O (Sigma-Aldrich) (KIT-6-Ni5-Y*x*) was used to get the fixed amount of nickel (5 wt %), and different content of yttrium in each sample (3, 6, or 8 wt %). After mixing the support with the metal precursor for 24 h, the slurry was transferred to a rotary evaporator with 110 rpm and a water bath set at 60 °C for removing the solvent excess. This step lasted 25 min. Afterward, the solid was dried overnight at 70 °C and calcined at 600 °C for 6 h (heating rate of 2.5 °C/min). The above-described preparation steps can be illustrated as shown in Scheme S1 (Supporting Information).

### 2.2 Characterization Methods

#### 2.2.1 Inductively Coupled Plasma–Optical Emission Spectrometry

Elemental analysis was performed with the aid of an ICP-OES 5110 Agilent VDV at Institut de Chimie des Milieux et Matériaux de Poitiers in France. Prior to the analyses, the samples were mineralized in an Anton-Paar Multiwave Pro microwave oven. The following mixtures of acids were used for mineralization steps: (i) 4 mL of HNO_3_ (>68%), 3 mL of HCl (34–37%), 1 mL of HF (47–51%) diluted in water, and (ii) 3 mL of H_3_BO_3_ (>68%) and 5 mL of HCl (34–37%) diluted in water.

#### 2.2.2 Nitrogen Physisorption

N_2_ adsorption–desorption of the calcined samples was carried out at −196 °C with a TriStar 3000 Micromeritics apparatus. The samples were previously outgassed under vacuum at 300 °C for 3 h^21^ by using a Micromeritics VacPrep 061 degas unit. The specific surface area (*S*_BET_) and cumulative volume of pores (*V*_p_) were calculated by the Brunauer–Emmett–Teller (BET) and Barrett–Joyner–Halenda (BJH) methods, respectively.

#### 2.2.3 X-ray Diffraction

XRD data were collected alone or together with XAS at BM31 of the Swiss-Norwegian beamlines (SNBL) at the European Synchrotron Radiation Facility (ESRF) in Grenoble, France. The data were collected with the aid of a 2D DEXELA detector using a Si(111) channel-cut monochromator set at a wavelength of 0.0338 nm. Quartz capillary reactors were provided by Hilgenberg GmbH and had a wall thickness of 0.01 mm, an outer diameter of 1.0 mm, and an overall length of 80 mm. Around 3–4 mg of the catalyst was placed between two plugs of quartz wool. The collected XRD data were averaged based on five images.

#### 2.2.4 Electron Microscopy

High-resolution transmission electron microscopy (HRTEM) and elemental mapping using energy-dispersive X-ray spectroscopy in scanning transmission electron microscopy (STEM-EDS) were used to determine the morphology of the catalysts. Both analyses were carried out on a JEOL JEM-2100 Plus microscope. To compute the particle size distribution of nickel in the reduced catalyst, the particle diameters were determined by using ImageJ software.

#### 2.2.5 Temperature-Programmed Reduction in H_2_

Temperature-programmed reduction (H_2_-TPR) was carried out on a BELCAT-M (BEL Japan) instrument. Samples (around 30 mg) were placed in a quartz reactor and pretreated in Ar at 300 °C for 60 min. Thereafter, the temperature was cooled down to 50 °C, and a mixture of 5% H_2_/Ar was introduced for 10 min to stabilize the signal of the thermal conductivity detector (TCD). Finally, the sample was heated with a heating rate of 10 °C/min from 50 to 750 °C in the same reductive gas mixture. The sample was kept at 750 °C for 90 min.

#### 2.2.6 H_2_ Chemisorption

Metal dispersion of the calcined catalysts was measured by H_2_ chemisorption at 40 °C using a Micromeritics ASAP 2020 instrument. Before the analysis, around 100 mg of the sample was reduced in pure H_2_ flow with a heating rate of 10 °C/min to 750 °C and kept at this temperature for 90 min. The sample was subsequently purged with He to desorb H_2_ on the surface, and the temperature was reduced to 40 °C at a rate of 10 °C/min. Volumetric chemisorption was performed at 40 °C by periodically injecting pure H_2_ over the reduced catalyst. Nickel dispersion was determined according to the quantity of hydrogen uptake, assuming an adsorption stoichiometry of H/Ni = 1.^21,22^

#### 2.2.7 Temperature-Programmed Desorption of CO_2_

The basicity of the reduced catalyst was examined by a CO_2_-TPD technique performed in the same apparatus as H_2_-TPR. Once the sample was analyzed by H_2_-TPR, the temperature was cooled to 80 °C in He flow. Subsequently, CO_2_ adsorption was carried out by flowing a mixture of 10% CO_2_/He (50 mL/min, 60 min), and the sample was subjected to He flow for 30 min to desorb the physically adsorbed probe molecules. Finally, the temperature-programmed desorption was conducted in a flow of helium (50 mL/min) in the temperature range of 80–750 °C at a heating rate of 10 °C/min.

#### 2.2.8 In Situ XAS–XRD Experiments

XAS–XRD measurements were performed at the Swiss-Norwegian beamlines (SNBL, BM31) at the ESRF, France. XAS data were collected in transmission mode at the Ni K-edge. The monochromator was equipped with Si(111) double-crystals. For processing, analysis, and fitting of XAS data, the Demeter software suite was used. The Athena software was utilized for data processing, while the Artemis software was used for shell fitting. A metallic nickel standard foil was measured and used for energy calibration and alignment of the respective absorption edge. Operando XAS–XRD of excess-methane dry reforming over KIT-6-Ni and KIT-6-Ni-Yx catalysts (size fraction of 53 to 90 μm) assumed the following steps: (i) staying at 50 °C in He, (ii) heating up to 750 °C in a mixture of 4%H_2_/He (1 bar, total flow of ca. 4 mL/min) with a ramp of 10 °C/min at 1 bar, (iii) staying at 750 °C in a mixture of 4% H_2_/He (1 bar, total flow of ca. 4 mL/min) for 90 min, (iv) cooling down in He until 700 °C, (v) subsequently introducing a flow of excess-methane dry reforming mixture for 45 min (700 °C, 1 bar, total flow of ca. 4 mL/min), and then, (vi) final cooling of the sample in He to 50 °C. The detailed procedure containing XAS–XRD steps and experimental flowsheet are shown in the Supporting Information, Figures S1 and S2.

#### 2.2.9 Thermogravimetric Analysis

The amount of deposited carbon on the spent catalysts collected after the catalytic reaction was measured by thermogravimetric analysis (TGA) on a TA Q5000 IR thermobalance. Around 20 mg of used catalyst was heated in synthetic air (flow 100 mL/min) starting from 35 to 800 °C with a heating rate of 10 °C/min.

### 2.3 Catalytic Dry Reforming of Methane

Excess-methane dry reforming was performed in a fixed-bed quartz reactor at an atmospheric pressure. The samples were sieved into the size fraction of 53 to 90 μm. Before the reaction, the catalysts were reduced under 5% H_2_/Ar (50 mL/min) at 750 °C for 90 min and purged under Ar while decreasing the temperature to 700 °C. A mixture of over stoichiometric molar ratio of CH_4_/CO_2_/Ar = 5.3:3.0:1.7 with CH_4_:CO_2_ = 1.77 was used for the DRM at 700 °C assuming WHSV = 120,000 mL h^–1^ g_cat_^–1^ (total flow rate of 100 mL/min). The reaction products were analyzed by gas chromatography (490 Varian Micro-GC) with a TCD. The equations presented in the Supporting Information were used for the calculations of CH_4_, CO_2_ conversions, H_2_/CO molar ratio, site time yield, and CH_4_ and CO_2_ consumption rates.

## 3 Results and Discussion

### 3.1 Catalyst Characterization

#### 3.1.1 Elemental Composition, Textural and Structural Properties

Inductively coupled plasma–optical emission spectrometry (ICP-OES) analyses were carried out to gain information on the actual contents of Si, Ni, and Y in the studied catalysts. The values are indicated in Table 1. After adding Y, no apparent difference was found with the targeted value of nickel showing ca. 5 wt % of this metal. The loading of yttrium changed as deliberately assumed to be 3, 6, and 8 wt %. This resulted in Y/Ni molar ratios of 0.5, 1.07, and 1.5 for KIT-6-Ni5-Y3, KIT-6-Ni5-Y6, and KIT-6-Ni5-Y8, respectively.

**Table 1:** Elemental Composition, Textural and Structural Parameters, and Ni Particle Size of the Catalysts

	elemental composition [wt %]^a^					Ni particle size [nm]^d^					
catalyst	Si	Ni	Y	*S*_BET_ [m^2^/g]^b^	*V*_p_ [cm^3^/g]^c^	XRD: when reached 750°C^d^	XRD: 90 min at 750°C^d^	TEM^e^	H_2_ chemisorption^f^	*D*_Ni_ [%]^g^	H_2_ consumption [mmol/g]^h^
KIT-6	n/a	n/a	n/a	733	0.88					n/a	
KIT-6-Ni5	27.7	6.2	<0.12	640	0.81	11.8	12.1	13	12	8.4	1.03 (1.05)
KIT-6–Ni5-Y3	33.4	5.1	2.6	560	0.69	11.3	12.1	23	19	5.2	0.80 (0.87)
KIT-6–Ni5-Y6	30.0	5.5	5.9	498	0.64	12.0	14.5	40	65	1.6	0.92 (0.94)
KIT-6–Ni5-Y8	31.1	5.3	8.1	465	0.62	12.8	16.7	59	110	0.9	0.90 (0.91)

a Estimated by ICP-OES.

b Calculated by the BET method.

c Calculated from BJH desorption cumulative volume.

d Calculated from 2θ = 18.3° (no overlapping peaks of carbon and nickel phases) by using the Scherrer equation ; *K* = 0.94 for spherical nickel crystallites with cubic symmetry, β is the full width at half-maximum (*FWHM*) of the peak, and θ is the Bragg angle.

e Assessed based on statistically estimated particle sizes which were measured by the ImageJ program.

f Assuming uniform spherical particles of nickel.

g Exposed metal fraction to total Ni estimated by H_2_ chemisorption (H/Ni = 1).

h Theoretical H_2_ consumption for Ni species is 0.85 mmol/g assuming metal loading of 5 wt % (in the parentheses are given theoretical values based on the metal loading detected by ICP-OES).^23^

**Figure 1:**
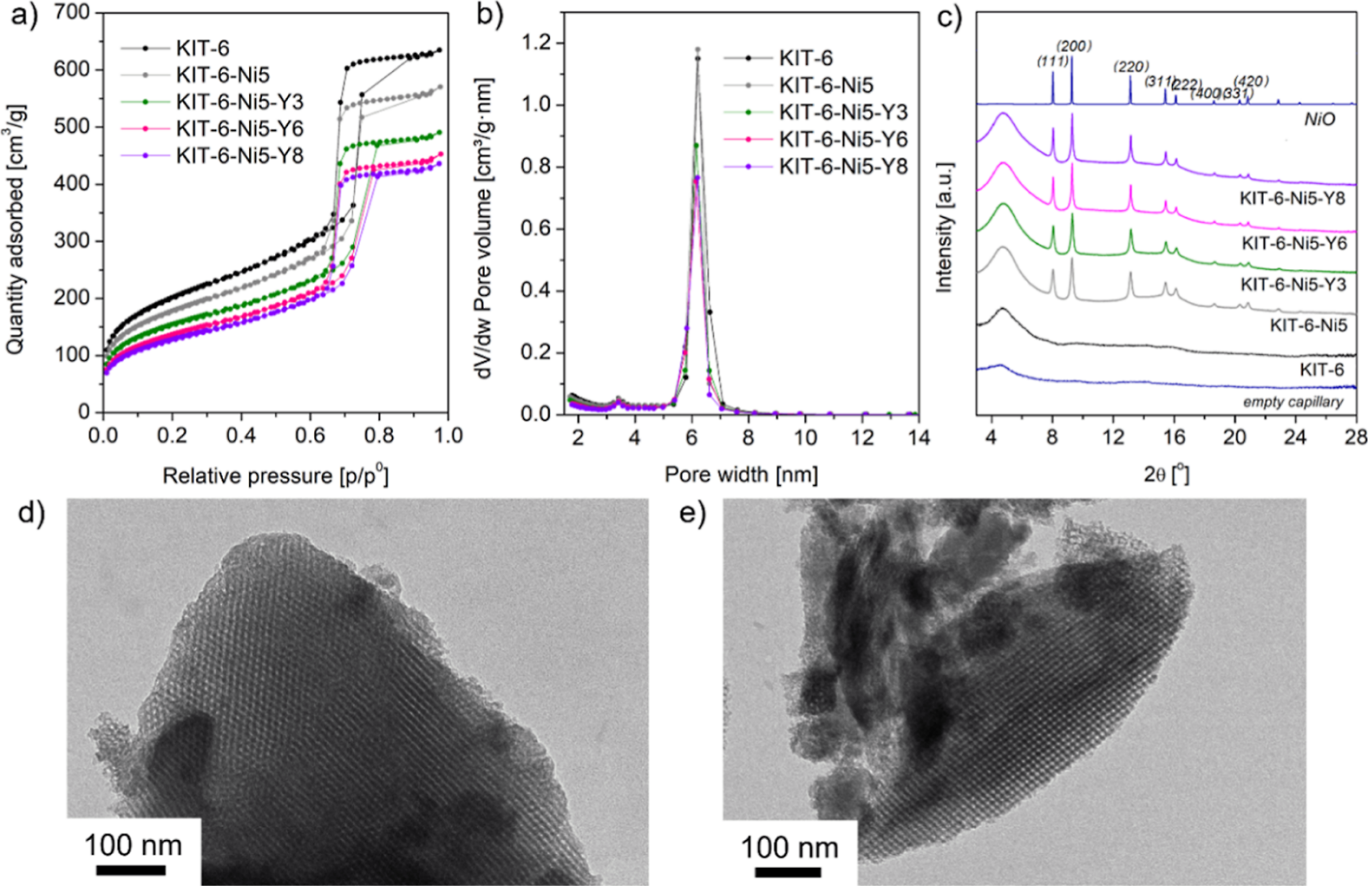
N_2_ adsorption–desorption isotherms of the calcined KIT-6 support and Ni, Y-containing catalysts (a), pore size distribution (b), X-ray diffractograms collected for the calcined samples (c), and TEM micrographs of the synthesized KIT-6 support (d,e).

N_2_ adsorption–desorption isotherms of calcined KIT-6 and its modified materials are presented in Figure 1a. According to the IUPAC classification, all the samples exhibited type-IV isothermal curves along with a sharp capillary condensation step and H1 hysteresis loops in the (*p*/*p*^0^) range of 0.69 to 0.75–0.80.^24^ This indicates that the mesoporous structure is possessed with large channel-like pores and narrow pores, which can be also verified by the well-defined pore size of ca. 6 nm in the corresponding Figure 1b. After modification with Ni, the height of the hysteresis loop decreased compared to KIT-6, whereas the width of the hysteresis loops remarkably increased after yttrium addition. The former can be linked with a decrease in pore volume, whereas the expansion of the hysteresis can be due to the change in the structure of pores caused by metal loading.^25^ The detailed textural parameters are given in Table 1. The bare KIT-6 support exhibited a relatively large specific surface area of 733 m^2^/g, as well as a high pore volume of 0.88 cm^3^/g. The calculated *S*_BET_ matches well with the specific surface area of KIT-6-100 (synthesized at 100 °C; 763 m^2^/g) described in the study of Zhou et al.^26^ The *S*_BET_ and pore volume of the KIT-6-Ni-Y catalyst remarkably declined with increasing yttrium loading, suggesting that the yttrium species partially blocked the channels of the silica support. At the same time, the high yttrium level could accelerate the aggregation of Y species located on the outer catalyst surface, thereby being able to block the pore structure. Nevertheless, the highest loading of yttrium still achieved a relatively high *S*_BET_ of 465 m^2^/g and a pore volume of 0.62 cm^3^/g.

The wide-angle XRD patterns of the calcined catalysts are shown in Figure 1c. All the catalysts showed a broad peak at 2θ = 4.8°, originating from the amorphous SiO_2_ in the KIT-6. This settles a negligible contribution of the X-ray scattering coming from the empty capillary. The diffraction peaks located at 2θ = 8.1, 9.3, 13.2, 15.5, 16.2, 18.7, 20.3, and 20.9° were, respectively, assigned to the crystal planes of (111), (200), (220), (311), (222), (400), (331), and (420) belonging to crystalline NiO (matching well with the standard).^27,28^ Similarly to our previous studies on Ni–Y mesoporous silica catalysts,^13,29^ no segregate diffraction peaks were detected for yttrium species. This suggests either high dispersion over the KIT-6 matrix or an amorphous character.

To further confirm the successful synthesis of the KIT-6 support, microscopy analysis was carried out. Figure 1d,e shows representative HRTEM micrographs with the evident highly ordered structure. The average pore size diameter was estimated to be 5–7 nm, which is in good agreement with the N_2_ physisorption results.

#### 3.1.2 Reducibility of KIT-6-Ni and KIT-6-Ni-Y Catalysts

**Figure 2:**
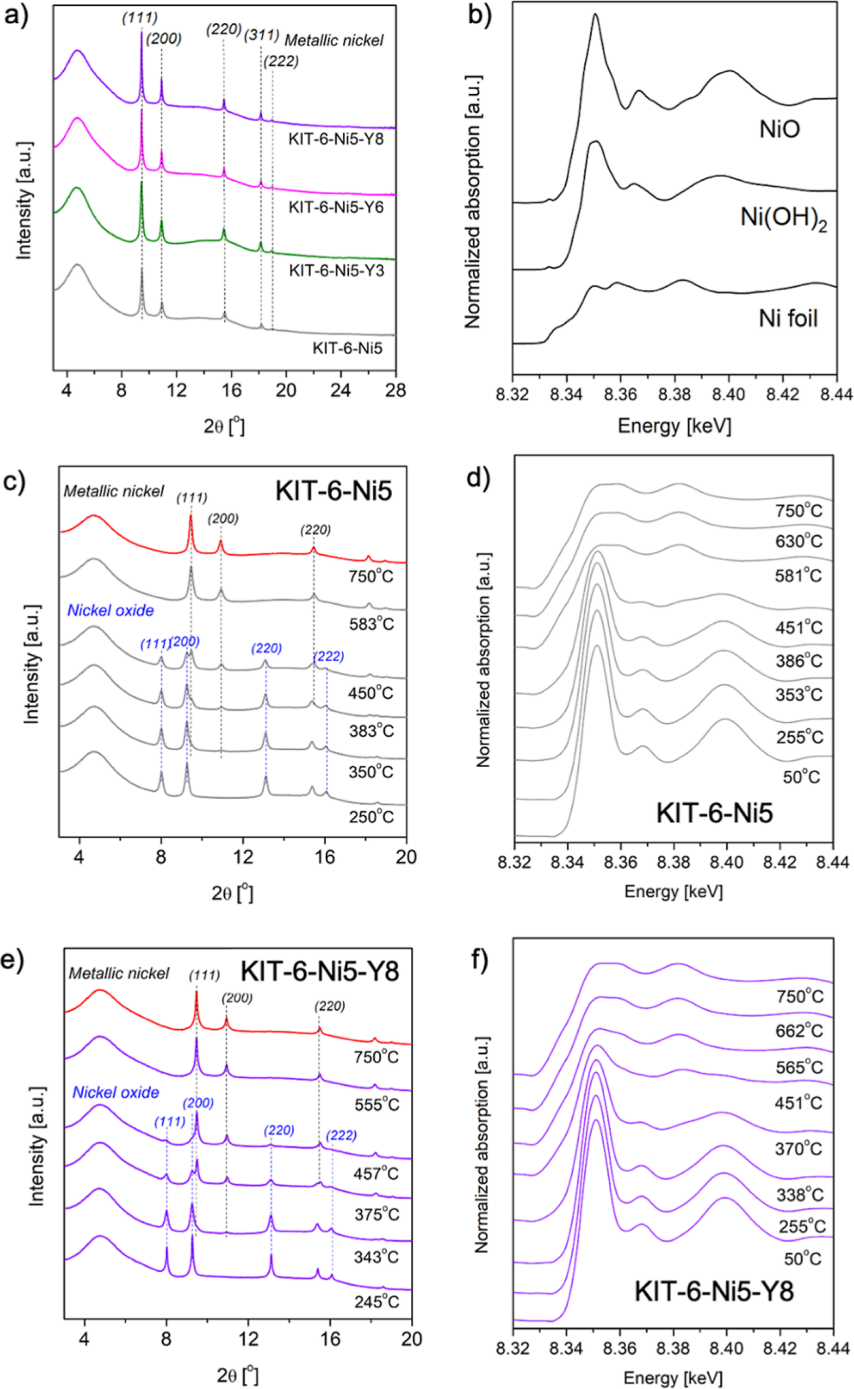
Combined in situ XRD–XAS during reduction: XRD patterns of the catalysts collected during in situ reduction (wavelength of 0.0338 nm, reduction condition: 4% H_2_/He, heating from 50 to 750 °C with a heating rate of 10 °C/min and kept for 90 min at this temperature); (a) diffractograms collected for the reduced samples after 90 min at 750 °C, (b) in situ Ni K-edge XANES with NiO, Ni(OH)_2_, and Ni foil standards, (c,e) in situ XRD patterns collected for KIT-6-Ni5 and KIT-6-Ni-Y8 catalysts collected during heating at different temperatures from 245 to 750 °C, and (d,f) in situ Ni K-edge XANES collected at different temperatures from 50 to 750 °C for KIT-6-Ni5 and KIT-6-Ni5-Y8.

In situ XAS–XRD during reduction was carried out to obtain a more comprehensive understanding of the reducibility of Y-modified KIT-6-Ni catalysts. Figure 2a,c,e presents diffractograms of the samples treated in a mixture of 4% H_2_/He at 750 °C after 90 min. All the samples showed a broad peak of mesoporous silica at 2θ = 4.8°, as well as reflections of the metallic nickel phase [2θ = 9.5, 11.0, 15.6, 18.3, 19.1° corresponding to (111), (200), (220), (311), (222) planes, respectively]. No peaks from yttrium-containing phases were observed which may be related to the high dispersion of yttrium species or their amorphous character. The Scherrer equation was used to calculate the Ni crystallite size, and the estimated values are listed in Table 1. One can note that reduction at 750 °C led to the creation of crystallites with a size of ca. 12 nm in all the studied samples. Promotion with 6 and 8 wt % of yttrium resulted in increased size of nickel crystallites after keeping the materials at 750 °C for 90 min (14.5 nm for KIT-6-Ni5-Y6 and 16.7 nm for KIT-6-Ni5-Y8). The growth of the supported nickel nanoparticles is a result of thermal sintering, also called migration and coalescence. Keeping the catalysts for a longer time at high temperature favored mobility of the Ni atoms being able to migrate and increase in size. The thermal sintering was not evident in KIT-6-Ni5 and KIT-6-Ni5-Y3 catalysts (no change in Ni particle size). According to the results from in situ XRD, NiO completely disappeared in favor of Ni^0^ at 583 °C for KIT-6-Ni5, while the same transformation was possible at 555 °C for KIT-6-Ni5-Y8. Figure 2d,f represents synchrotron XANES spectra for the Ni K-edge of KIT-6-Ni5 and KIT-6-Ni5-Y8, respectively. All the recorded XANES spectra are presented in Figure S3. The similarity with the nickel foil (shown in Figure 2b) implies a reduction degree of Ni close to 100%. In the current study, only small differences can be observed between the XANES of the Ni foil standard and the spectra recorded for our catalysts which can arise from the fact that in our samples nickel nanoparticles are deposited on a support. Ni(OH)_2_ was not detected when increasing the temperature from 50 to 750 °C. Nickel nitrate, used as a precursor during catalyst synthesis, is known to be decomposed into NiO before being reduced at temperatures of ca. 500–600 °C.^30^

**Figure 3:**
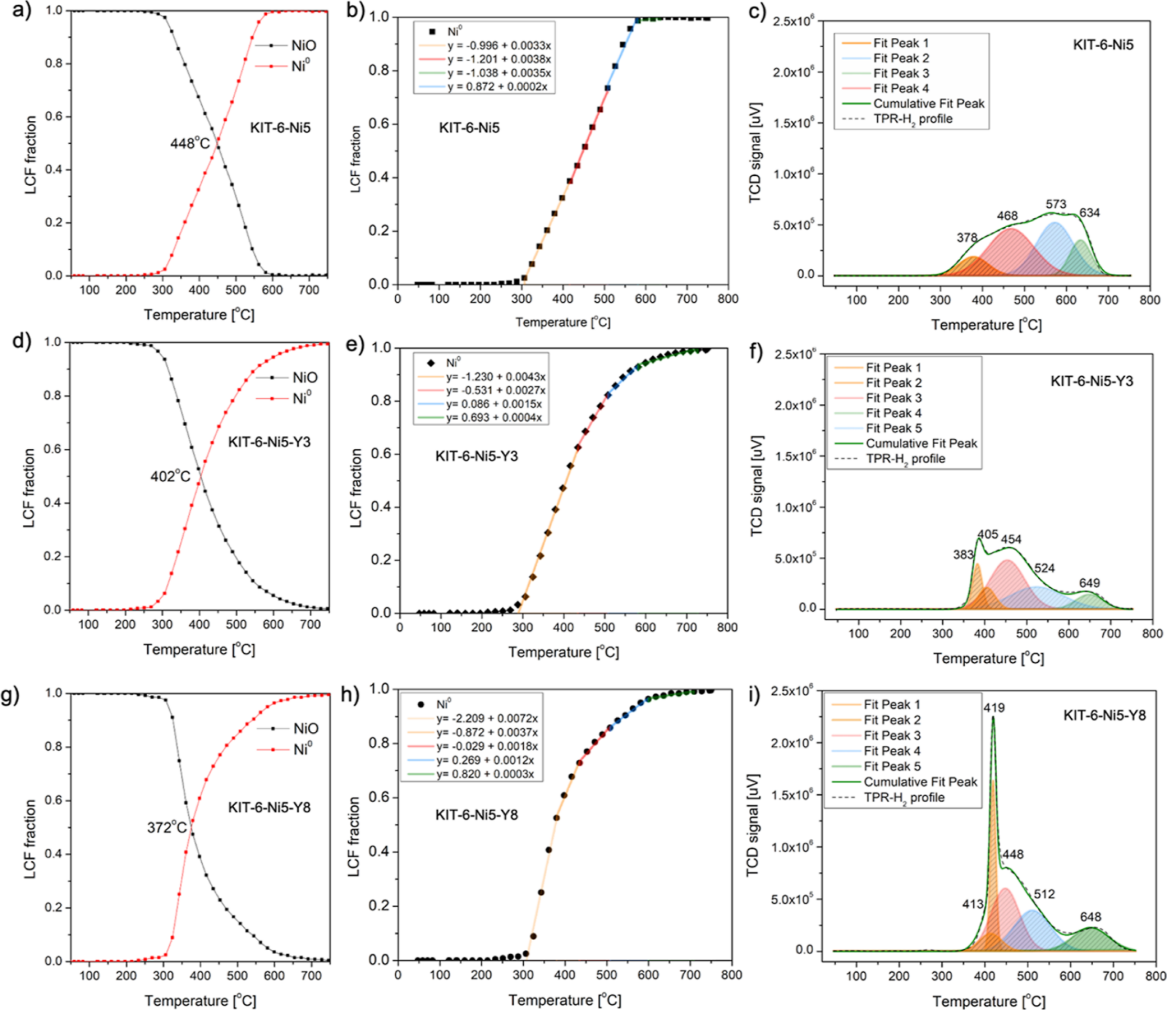
LCF of XANES (a,d,g), LCF fraction against temperature for estimation of the rate of reduction (b,e,h), and H_2_-TPR profiles of KIT-6-Ni5, KIT-6-Ni5-Y3 and KIT-6-Ni5-Y8 catalysts (c,f,i).

LCFs estimated for the temperature-programmed reduction are presented in Figure 3a,d,g for the unpromoted sample and with the lowest and highest content of yttrium, respectively. The in situ results confirmed the full reduction of NiO to Ni^0^ at a certain temperature, which turned out to be influenced by the presence of yttrium. Yttrium allowed metallic nickel to appear at a much lower temperature compared to the unmodified sample (in agreement with XRD described above), thereby improving the reducibility of Ni species. The reduction temperature of NiO decreased with increasing yttrium content (402 °C for KIT-6-Ni5-Y3 and 372 °C for KIT-6-Ni5-Y8). However, the facilitated reducibility had only a slight impact on the final degree of reduction. In other words, at the temperature of 750 °C metallic nickel was the dominating phase in all the studied samples. The collected data points allowed us to identify a sequence of different reduction rates of nickel oxide. Here, we propose the following understanding in which different forms of nickel can be identified by trend lines (having different slopes) found in the XANES LCF figure. A scenario assuming one curve going through all the data points is presented in Figure S4a–c for KIT-6-Ni5, KIT-6-Ni5-Y3, and KIT-6-Ni5-Y8, respectively. One can note that the trend lines are not completely linear in the entire temperature range, which may be due to reduction of different nickel species. A linear law of reduction should be applied; thus, a series of linear fittings from 0 fraction to 1.0 was assumed. Four trend lines were distinguished (Figure 3b,e,h) and were marked in orange, red, blue, and green referring, respectively, to bulk NiO, weakly interacting, strongly interacting with the support, and NiO located inside the support channels. For the unpromoted catalyst, the rates seem to be alike, which contrasts with those of the yttrium-modified samples. For the Y-series, the steepest trend lines appeared at the lowest temperatures, i.e., the yellow color lines present below 450 °C. These refer to the fastest reaction rates. For the KIT-6-Ni5-Y8 sample, this stage has been divided into two steps (300–380 and 380–440 °C), possibly due to diverse interactions with the support or a significant variance in terms of the crystallite size. Less steep curves emerge at higher temperatures, finally reaching a relatively flat curve above 600 °C (green color line). It should be stressed that these results can provide only a qualitative estimation of the reduction rate of different species. The analysis is not quantitative. Moreover, the collected data points do not allow us to describe all processes involved in the reduction, e.g., shrinking core with nonlinear mechanisms. A direct correlation between the estimated trend lines and H_2_-TPR data can then be proposed. H_2_-TPR of KIT-6-Ni5 (Figure 3c) revealed four wide peaks at temperatures between 300 and 700 °C which can be attributed to the reduction of both bulk nickel oxide located on the outer surface weakly interacting with the support (low temperature peak at ca. 450 °C) and reduction of strongly interacting with the carrier NiO and more dispersed small crystallites (high temperature peaks at temperatures between 500 and 750 °C).^15,31,32^ In the yttrium-containing samples, a sharp peak centered at ca. 400 °C was registered and is associated with increased reducibility of large crystallites of bulk nickel oxide interacting with the silica support. Ni^2+^ species, which are much more difficult to reduce, need a higher temperature (peaked at 512 and 648 °C) and therefore have slower reduction rates. Mile et al.^31^ studied the location of nickel oxide during H_2_ reduction in silica-supported catalysts. The authors emphasized that morphological factors are as important as topological properties in determining the course of reduction. In the current study, yttrium promotion clearly changed the surface of the nickel-loaded KIT-6, causing nickel species to fall outside the channels of silica and form larger particles weakly interacting with the support and consequently leading to increased reduction rates, as reported in Figure 3e,h. On the other hand, Parravano^32^ studied NiO reduction together with different foreign ions (Ag^+^, Li^+^, Mg^2+^, Cr^3+^, etc.). It was observed that in almost all cases, the rate of reduction decreased. Yttrium was not the subject of his study. Analyzing the results of the current work, one can see that in some temperature zones (colored in red and blue), the reduction rates are lower than for the unpromoted sample. Moreover, a recent study by Acharya et al.^33^ showed a successful capture of reduction of nickel in bimetallic Ni–Fe catalysts during operando electrocatalytic reaction monitored by XAS. Due to the short acquisition time, which is not the case in our study, the authors were able to study kinetics by using LCF analysis for time-resolved changes. First-order reaction rates were obtained for their samples. Moreover, the LCF analysis showed different predictions in the relative amounts of contributing phases, suggesting that the time-resolved operando XAS was crucial to fully understand the changes in phase fraction for their complex electrocatalyst materials.

H_2_-TPR profiles of the studied catalysts can be found in Figure S5. Clearly, ramping up to temperatures equal to and above 750 °C is one of the possible requirements to fully reduce nickel oxide phases. A comparison between the theoretical H_2_ consumption value and calculated results from our samples confirmed the full reducibility of the studied samples (Table 1). It is well known that reducibility of nickel largely depends on the dispersion of Ni, as well as Ni interactions with the support.^34^ According to Bellido and Assaf,^35^ the reduction of Y_2_O_3_ can be neglected, as only 0.24% of pure Y_2_O_3_ is reducible at ca. 650 °C. Hence, the peaks registered in our H_2_-TPR patterns were attributed to the reduction of nickel species, although some NiO-Y_2_O_3_ interactions can still be defined. The presence of yttrium seems to alter the nickel reducibility, as a systematic shift of high-temperature reduction peaks (at ca. 650 °C) to lower temperatures with increasing yttrium content can be observed. For KIT-6-Ni5-Y8, this can indicate stronger interactions between NiO and the support or improved dispersion of nickel particles placed inside the three-dimensional pore network of KIT-6. Nevertheless, in each yttrium-promoted sample, the sharp peak (at ca. 400 °C) becomes more pronounced with increasing metal loading. The increased size of the crystallites can be a main factor explaining the growing intensity of this single peak.^34^ It appears that KIT-6-Ni5-Y8 characterizes very strong interactions between small Ni particles and the support, as well as the presence of relatively large Ni particles which are outside the support channels. In this case, the shift in reduction temperature is not significant with increasing the content of yttrium. Moreover, it is important to mention possible interactions between NiO and Y_2_O_3_. The existence of the NiYO_3_ phase is feasible and can be explained by the diffusion of Ni into the lattice of Y_2_O_3_ at ca. 720 °C due to the smaller size of Ni^2+^ ions (0.078 nm) than Y^3+^ ions (0.089 nm).^36,37^

**Figure 4:**
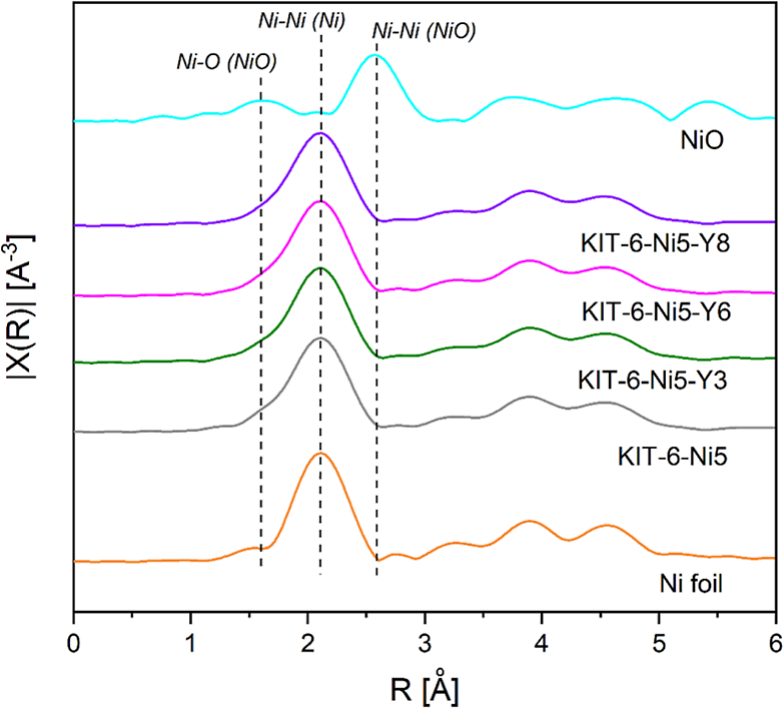
Ni K-edge Fourier-transformed *k*^3^-weighted EXAFS of the reduced catalysts along with Ni foil and NiO as the references (not phase corrected).

The extended X-ray absorption fine structure (EXAFS) at the Ni K-edge was examined to obtain detailed structural information about the nickel active phase. The *k*^3^-weighted Fourier-transformed EXAFS spectra and standards were plotted in R-space and *k*-space and presented in Figures S6–S8 (Supporting Information). The NiO standard consisted of two main paths with radial distances of ca. 2.1 and 2.9 Å, which can be assigned to Ni–O and Ni–Ni pairs, respectively. Nickel foil (used as a standard for metallic nickel) showed a characteristic Ni–Ni bond distance at 2.5 Å. The structural parameters of the standards are in line with the previously reported results.^10,38,39^ Upon reduction of the catalysts, the Ni–Ni peak (at a distance of ca. 2.5 Å) became as intense as the corresponding peak from Ni foil with a coordination number close to 12. When comparing the collected spectra presented in Figure 4, one can see that KIT-6-supported catalysts exhibited another scattering path identified as Ni–O at ca. 2.1 Å and appeared as a leftward shoulder of the most intense peak revealed in EXAFS. However, this speculation would lead us to believe that some parts of the catalysts remained unreduced which contradicts the results of H_2_-TPR and XANES. The fit of the second shell (data not shown) confirmed the absence of the Ni–Ni bond originating from nickel oxide; therefore, the studied materials contained only a reduced phase. Moreover, *k*^3^-weighted Fourier-transformed EXAFS spectra plotted in *k*-space resembled that of Ni foil (Figure S8). It should be noted that the EXAFS spectra were recorded at 750 °C for our samples, whereas the standards were collected at room temperature. The temperature difference led to a less satisfactory fit of the spectra and higher R-factors. Table 2 summarizes bond distances, coordination numbers, Debye–Waller type factors, standard deviations, and *R*-factors. The Ni–Ni coordination numbers were fixed to 12.0 to match the measured sizes of Ni crystallites.

**Table 2:** Structural Parameters Obtained by EXAFS Fitting (Ni K-edge) for the Standards and Reduced KIT-6-Ni5-Y*x* Catalysts

sample	state	bond	coordination number	*R* [Å]	σ^2^ [Å^2^]^b^	*R*-factor
NiO	standard	Ni–O	6.0 (fixed)	2.1 ± 0.01	0.008 ± 0.0003	0.009
	standard	Ni–Ni	12.0 (fixed)	2.9 ± 0.02	0.008 ± 0.0012	
Ni foil	standard	Ni–Ni	12.0 (fixed)	2.5 ± 0.01	0.006 ± 0.0003	0.008
KIT-6-Ni5	reduced^a^	Ni–Ni	12.0 ± 0.1	2.5 ± 0.01	0.008 ± 0.001	0.023
KIT-6–Ni5-Y3	reduced^a^	Ni–Ni	12.0 ± 0.1	2.5 ± 0.01	0.009 ± 0.001	0.020
KIT-6–Ni5-Y6	reduced^a^	Ni–Ni	12.0 ± 0.1	2.5 ± 0.01	0.008 ± 0.001	0.024
KIT-6–Ni5-Y8	reduced^a^	Ni–Ni	12.0 ± 0.1	2.5 ± 0.01	0.008 ± 0.001	0.026

a Reduced in a mixture of 4% H_2_/He at 750 °C for 90 min.

b Debye–Waller type factor.

**Figure 5:**
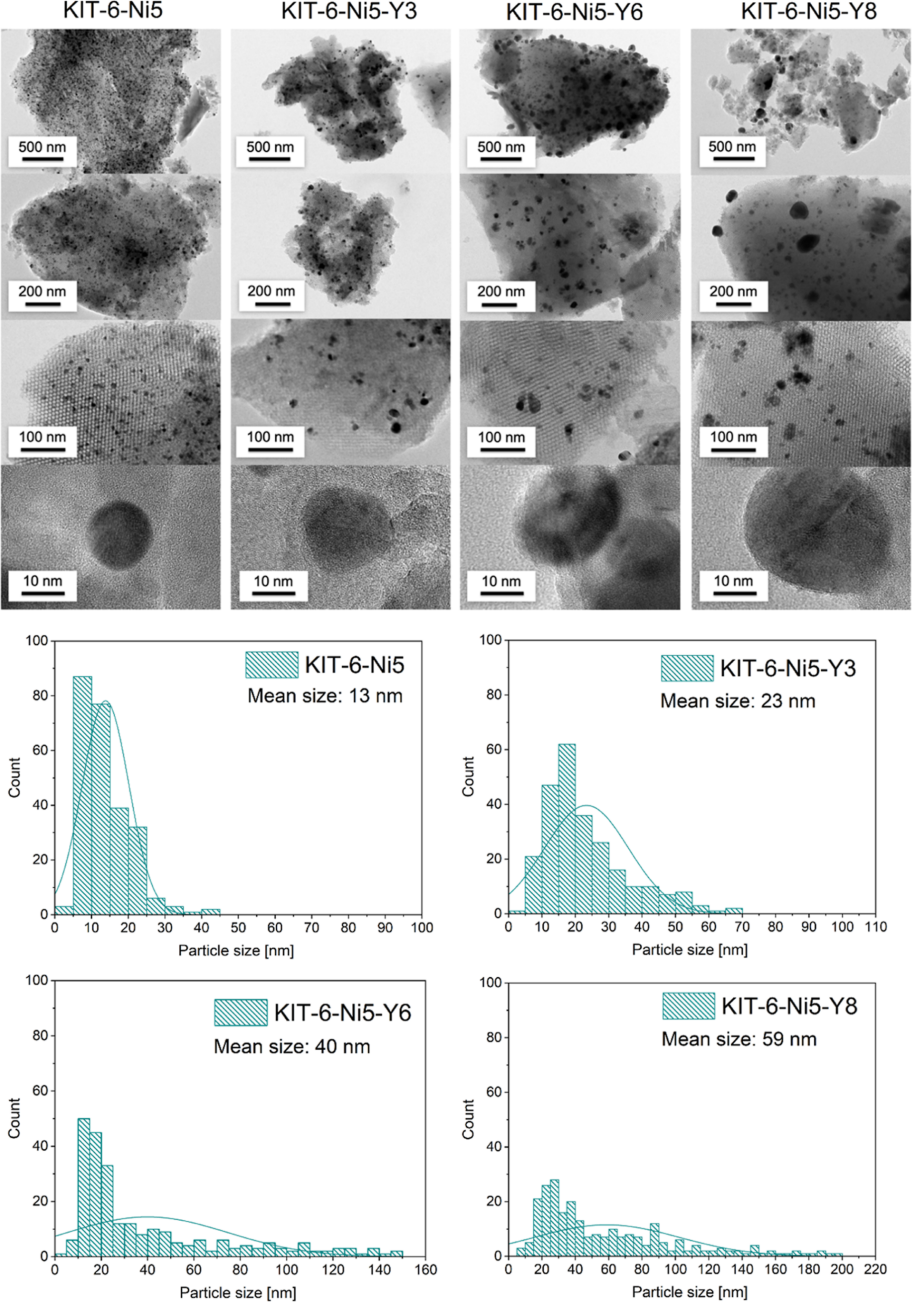
HRTEM images with the corresponding histograms of the reduced catalysts.

Microscopy analysis allowed us to image the supported nanoparticles on the surface of the reduced catalysts. After H_2_ treatment at 750 °C, KIT-6 support maintained an ordered mesoporous structure (Figure 5). The micrographs demonstrated randomly dispersed hemispherical particles with variable sizes of even smaller than 10 nm. The unpromoted catalyst contained highly dispersed small nickel particles, which were located inside the KIT-6 pores and on the outer surface. The histograms in Figure 5 clearly evidenced an increase in mean Ni particle size with increasing yttrium loading from 13 nm for KIT-6-Ni5 to 23, 40, and 59 nm for KIT-6-Ni5-Y3, KIT-6-Ni5-Y6, and KIT-6-Ni5-Y8, respectively. Although significantly larger particles were found in the Y-modified samples, there were still many particles smaller than 30 nm. The reported increase in particle size is consistent with the XRD and H_2_ chemisorption analyses.

**Figure 6:**
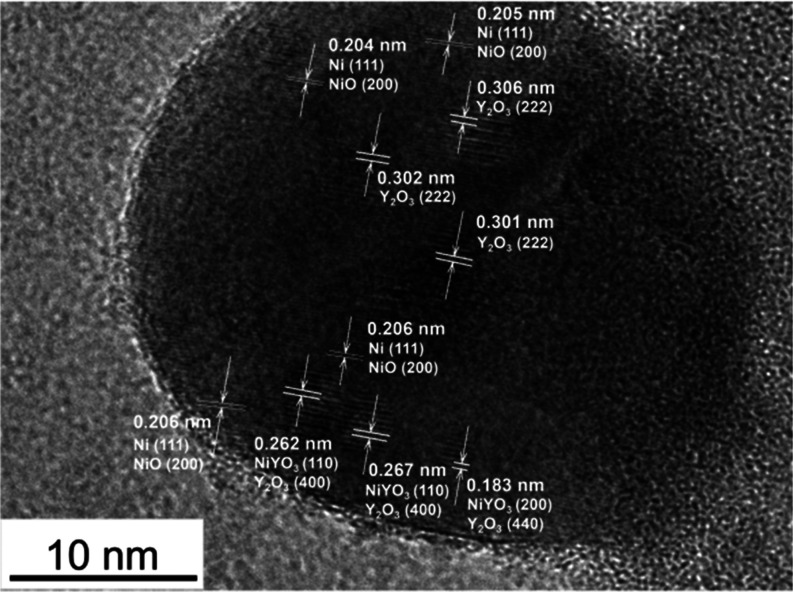
HRTEM micrograph of a particle in the reduced KIT-6–Ni5-Y8 catalyst.

The elemental distribution of Si, O, Ni, and Y was determined by STEM-EDS elemental mapping (Figure S9 in Supporting Information). Note that spherical particles of nickel are clearly visible, whereas yttrium appears to be highly dispersed over the support as well as on the spots where nickel particles are located. This indicates the proximity between Ni and Y after reduction. The diffraction analysis performed on the dark particles captured in the micrographs in Figure 6 predominantly showed the contribution of reduced nickel (Ni^0^ with interplanar spacing of around 0.20 nm). A minor presence of NiO is discerned and can be linked with sample passivation, as HRTEM was not performed in situ. A detailed HRTEM analysis of the catalyst with the highest amount of yttrium allowed us to record other fringes of the nanoparticles located on the outer surface of large Ni particles (Figure 6). The measured d-spacings revealed distances of 0.183, 0.262, 0.267, 0.301, 0.302, and 0.306 nm that can originate from Y_2_O_3_ or NiYO_3_. Thus, it appears that Y-containing particles decorate the metallic Ni particles. The high dispersion of yttrium species is supported by the in situ XRD results, in which no diffraction peaks were found for yttrium-containing species.

H_2_ chemisorption was employed to estimate the dispersion of nickel. As summarized in Table 1, the dispersion decreased in the following order: KIT-6-Ni5 (8.4%) > KIT-6-Ni5-Y3 (5.2%) > KIT-6-Ni5-Y6 (1.6%) > KIT-6-Ni5-Y8 (0.9%) in correlation with the obtained *S*_BET_ (Figure S10 in Supporting Information). The size of Ni particles assessed from the HRTEM analyses is in fair agreement with the results of H_2_ chemisorption. The yttrium-promoted samples revealed exceptionally large particles (<200 nm) which can explain the low average nickel dispersion found by H_2_ chemisorption. Moreover, as shown in the micrographs, yttrium covered part of the Ni surface, leading to a certain underestimation of the measured sizes. An inconsistency in the particle size may be found with respect to the XRD results. The observed differences between the estimations made by H_2_ chemisorption, TEM, and XRD can arise from the nature of nickel particles.^40^ It is likely that several small nickel particles are agglomerated. The agglomerate can appear as one large particle in the micrographs (Figure S11 in Supporting Information). XRD data give information on the crystalline particles, while microscopy analysis is not that detailed in terms of estimation of each particle size. Accordingly, each characterization technique has certain limitations in the calculation of average nickel particle size.^41,42^

#### 3.1.3 Basicity of KIT-6-Ni and KIT-6-Ni-Y Catalysts

**Figure 7:**
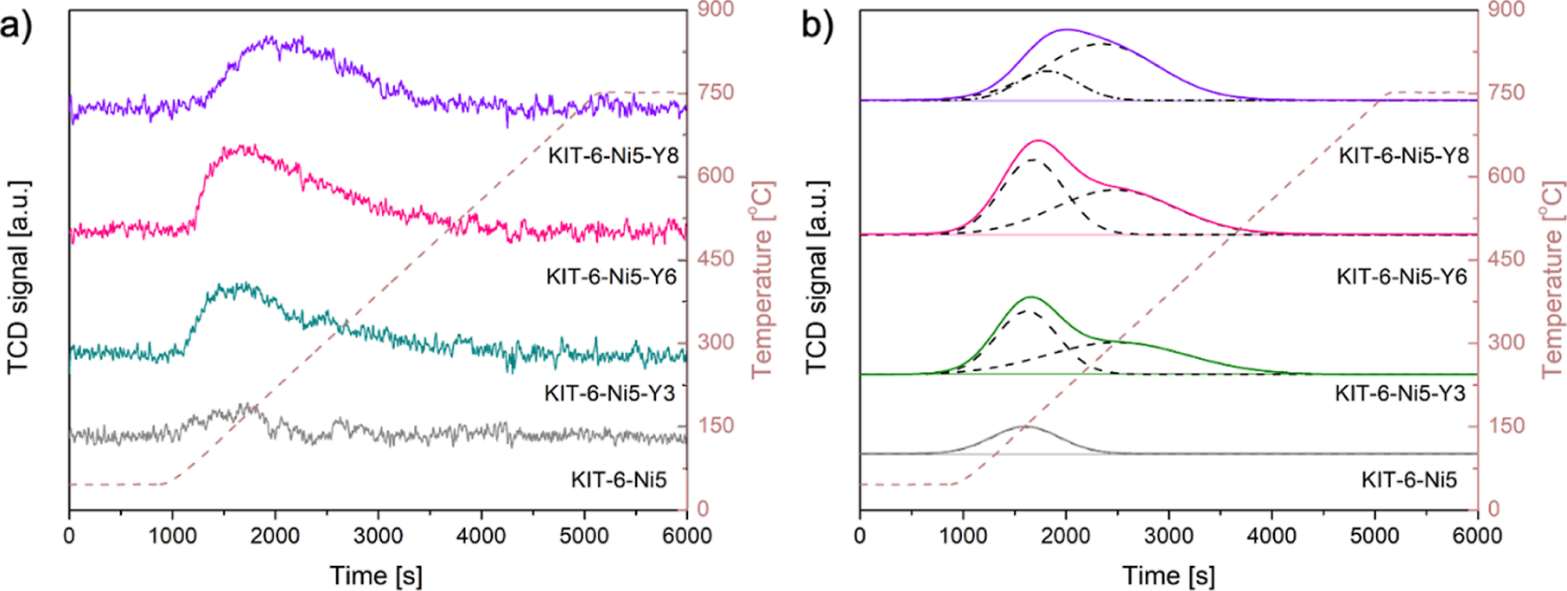
CO_2_ desorption profiles for the reduced KIT-6-Ni5-Y*x* catalysts (*x* = 3, 6, 8) (a), Gaussian deconvolution of the CO_2_-TPD peaks (b).

The above-mentioned redox properties of yttria-promoted samples may affect not only the reducibility but also the adsorption/desorption of CO_2_. Carbon dioxide, once adsorbed on the Y_2_O_3_ promoter, can refill the oxygen lattice.^43^ The mobility of oxygen is considered to be a positive surface feature that can contribute to the oxidation of accumulated carbon. It has been reported that surface basicity plays a crucial role in DRM. Not only because the accumulation of carbon can be reduced but also because the appropriate basicity endorses the activation and chemisorption of CO_2_, which is one of the reactants in the DRM reaction.^44^ To investigate the surface basicity of the reduced catalysts, temperature-programmed desorption with carbon dioxide as a probe molecule (CO_2_-TPD) was carried out. The registered profiles are shown in Figure 7a,b. The first peak located at ca. 85–220 °C refers to the weak basic sites originating from the bicarbonate species due to the interaction between hydroxyl species on the silica and adsorbed CO_2_ molecules.^29,45,46^ The second desorption peaks located between 220 and 500 °C were assigned to medium-strength basic sites regarded as metal–oxygen pairs.^45,47^ No peaks at temperatures higher than 500 °C were recorded, which are normally identified as strong basic sites. The values of the surface basicity are listed in Table 2. According to the results, the strong basic sites are restricted, while the amount of weak and medium basic sites are boosted when Y is added to the KIT-6-Ni5 catalyst. Higher content of Y (8 wt %) led to a decrease in weak basicity and an increase in medium-strength sites. Furthermore, the addition of yttrium enhanced the overall basicity, particularly in the case of 6 wt % of the metal loading. Increasing the total number of basic sites fundamentally means increasing the oxygen mobility that can later accelerate CO_2_ activation.^48^ It is not the first time that yttrium, used as a promoter, has been shown to increase the basicity. Some examples of different supported yttrium-promoted catalysts are given in Table S1. Wang et al.^49^ compared NiO–ZrO_*m*_–YO_*n*_ with NiO–ZrO_*m*_ in DRM. According to the authors, new weak basic sites formed upon modification with Y significantly enhanced the ability to eliminate carbon formation. The coke resistance was linked to the formation of surface carbonate species. This agrees with the study of Köck et al.^50^ who examined the adsorption of CO_2_ or CO on Y_2_O_3_ by in situ FT-IR. The authors identified the basic surface of Y-centers and suggested that CO_2_ molecules chemisorb onto reactive surface hydroxyl groups of Y_2_O_3_ forming bicarbonate species. According to Oemar et al.,^51^ yttrium oxycarbonate can be formed when CO_2_ is adsorbed on Y_2_O_3_ and reacts as follows: CO_2_ + Y_2_O_3_ = Y_2_O_2_CO_3_. The yttrium oxycarbonate can later react with the surface carbon to form CO and Y_2_O_3_. The efficiency of this reaction depends on whether the carbon removal rate from the oxycarbonate species is higher than the carbon deposition rate determined by CH_4_ decomposition and the Boudouard reaction. Another example of a yttrium-promoted catalyst is a Ni/Mg/Al hydrotalcite-derived catalyst (HTNi-Y1.5) tested in DRM^42^ (Table S1). The presence of yttrium led to an increase in the fraction of medium basic sites; however, the total basicity decreased after the addition of the promoter. An increase in medium-strength sites due to Y was also reported by Goma et al.^52^ and Battumur et al.^53^ in separate studies. However, in both reports, the total number of basic sites was comparable to that of other tested samples. In a study by Sun et al.^54^ on Ni/SBA-16 catalysts, total basicity increased with increasing yttrium loading up to 10 wt %. The addition of yttrium caused an increase in the number of medium basic sites, with a subsequent decrease in weak sites. XPS analysis revealed that yttrium led to the formation of Si–O–Y chemical bonds, which could account for the increase of moderate basic sites. Similar to our study, no strong basic sites were found in the Ni/Y/SBA-16 catalysts. It is known that in DRM, the optimal contribution of weak and medium basic sites for the activation of CO_2_ is significant.^48^

### 3.2 Dry Reforming of Methane

#### 3.2.1 Excess of Methane in the Gas Mixture

**Figure 8:**
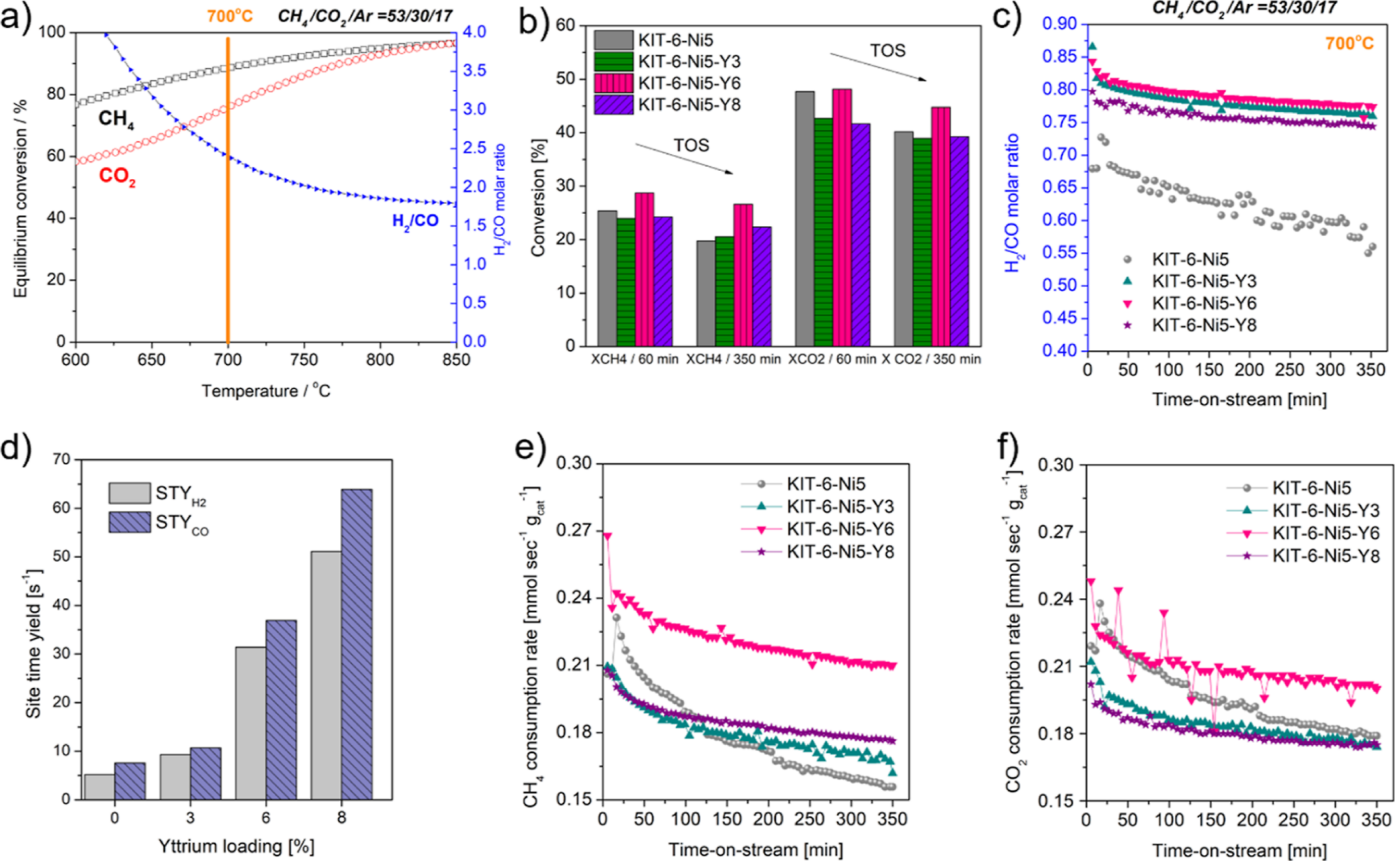
Results of excess-methane dry reforming including (a) thermodynamic equilibrium values estimated for CH_4_/CO_2_/Ar = 53:30:17 at a pressure of 1 bar, (b) initial conversions of CH_4_ (X_CH_4__) and CO_2_ (X_CO_2__) and the one obtained after 350 min, (c) H_2_/CO molar ratio registered over time-on-stream, (d) site time yield of H_2_ (STY_H_2__) and CO (STY_CO_), and (e,f) CH_4_ and CO_2_ consumption rates calculated in the time-on-stream.

Figure 8a–f presents catalytic results of the KIT-6-Ni5-Y*x* series, together with calculations of thermodynamic equilibrium values at 1 bar. A temperature of 700 °C was chosen, allowing the maximum conversion of 88.6% of CH_4_, and 75.8% of CO_2_ as well as a H_2_/CO molar ratio of 2.4 (Figure 8a). The tested catalysts revealed CH_4_ and CO_2_ conversion values lower than those of the thermodynamic equilibrium. The CO_2_ conversion was higher than the CH_4_ conversion for all catalysts. The former showed values below 50%, while the latter were below 30%. Methane conversion appeared to be low because of its surplus in the feed, similarly as reported in the studies of other authors.^55,56^ Among the tested samples, KIT-6-Ni5-Y6 had the highest activity with CH_4_ and CO_2_ conversions of 26 and 44%, respectively, after 350 min. This observation agrees with the results from our previous work on stoichiometrically conducted DRM. We reported that a certain loading of yttrium is particularly beneficial in DRM. A series of dry impregnated samples showed the following activity sequence at 700 °C: 12 wt % < 4 wt % < 0 wt % < 8 wt %, whereas in the current study (wet-impregnated series) the following ranking of initial activity was found: 8 wt % ≈ 3 wt % < 0 wt % < 6 wt %. The deactivation factor (DF) has been calculated for the wet-impregnated catalysts after a 350 min reaction. The results are listed in Table 4. The lowest DF has been observed for KIT-6-Ni5-Y6 (0.11), while the highest for the unpromoted catalyst (0.25). The DF was correlated with the total basicity of the reduced materials (Figure S12). It appears that yttrium led to the formation of new basic sites and that high total basicity accounts for more stable performance in excess-methane dry reforming. Moreover, yttrium promotion positively influenced selectivity, the obtained H_2_/CO ratio was stable in the range of 0.83–0.7, while the unpromoted catalyst showed decreasing values over time. The addition of yttrium also influenced the site time yield of H_2_ and CO (STY: number of molecules of a specified product made per catalytic site per unit time). The obtained values are listed in Table 4. The produced H_2_ was calculated from methane consumption. The STY increased with increasing content of yttrium, being the highest for KIT-6-Ni5-Y8. Yttrium-promoted samples showed a significantly lower number of moles of Ni active sites compared to KIT-6-Ni5 which can be directly linked with Ni dispersion.^57^ Small Ni crystallite size provides a high number of active sites, which was observed for the unpromoted sample (Table 3). The number of sites decreased with increasing loading of yttrium, i.e., increasing crystallite size of nickel.

**Table 3:** Basic Site Distribution of the Catalysts Derived from CO_2_-TPD

	basic sites [μmol/g_cat_]			distribution of basic sites [%]	
catalyst	weak	medium	total basic sites [μmol/g_cat_]	weak	medium
KIT-6-Ni5	16.0	0	16.0	100	0
KIT-6–Ni5-Y3	29.9	34.8	64.7	46.2	53.8
KIT-6–Ni5-Y6	40.0	45.5	85.6	46.8	53.2
KIT-6–Ni5-Y8	15.0	53.8	68.8	21.8	78.2

Consumption rates of CH_4_ and CO_2_ are presented in Figure 8e, f. In excess-methane dry reforming over KIT-6-Ni5-Y6 and KIT-6-Ni5-Y8, both gases were converted at approximately the same rate with consumption of CH_4_ only slightly higher than for CO_2_. When the other samples were tested at excess methane, larger amounts of CO_2_ were converted compared to CH_4_ (Table 4). Some fluctuations were recorded for carbon dioxide, which can be due to the coexistence of reactions producing and consuming CO_2_.

**Table 4:** Catalytic Results of DRM Carried Out in Excess of Methane, Including the DF, STY_H2_, and STY_CO_ (Site Time Yield), Number of Moles of the Ni Active Sites Calculated from the Dispersion Estimated by H_2_ Chemisorption, and Consumption Rates of CH_4_ and CO_2_ Registered for the Studied Catalysts

catalyst	DF^a^	STY_H_2__ [s^–1^]	STY_CO_ [s^–1^]	Ni sites [mol × 10^6^]	CH_4_ consumption rate [mol s^–^^1^·g_cat_^–^^1^]^b^	CO_2_ consumption rate [mol·s^–^^1^·g_cat_^–^^1^]^b^
KIT-6-Ni5	0.25	5.2	7.6	44.7	0.156	0.179
KIT-6–Ni5-Y3	0.22	9.3	10.7	22.6	0.162	0.174
KIT-6–Ni5-Y6	0.11	31.4	36.9	7.5	0.210	0.201
KIT-6–Ni5-Y8	0.14	51.1	63.9	4.0	0.176	0.175

a Deactivation factor = |X_CH_4_(350)_ – X_CH_4_(0)_|/X_CH_4_(0)_.

b Value obtained after 350 min of catalytic test.

Figure 8c presents the H_2_/CO molar ratio which can be directly linked to the differences in catalytic activity. In general, a H_2_/CO molar ratio lower or higher than unity can be explained by the presence of side reactions influencing the final amount of the produced hydrogen and carbon monoxide (DRM: 2CH_4_ + 2CO_2_ = H_2_ + CO). Since we use excess-methane conditions, this may not be straightforward. The studied catalysts clearly formed more CO than H_2_ and the obtained H_2_/CO molar ratio was significantly lower than that thermodynamically predicted (ca. 2.4 at 700 °C). This can be a consequence of the reverse water–gas shift (RWGS) reaction, leading to the production of excess CO and increased consumption of CO_2_.^55^ This appears to be the case for KIT-6-Ni5 and KIT-6-Ni5-Y3 catalysts, where CO_2_ consumption rates were considerably higher than for CH_4_. In principle, equal rates of CH_4_ and CO_2_ suggest that these molecules react with each other in a 1:1 ratio. When analyzing Figure 8c, one can see that the differences between CH_4_ and CO_2_ rates influence the stability of the H_2_/CO molar ratio. Moreover, another side reaction occurring under the applied conditions was a carbon-forming reaction. It was identified by a change in the appearance of the catalytic bed after the DRM. The gray-colored powder was found for all samples after the reduction step (e.g., following H_2_ chemisorption), and once the catalysts were used in the catalytic process, the powder changed color from gray to black, suggesting the formation of carbonaceous species. The presence of coke was also confirmed by TGA and HRTEM carried out on the spent catalysts. Reactions responsible for carbon formation are direct CH_4_ decomposition (DMD) and the Boudouard reaction given by CH_4_ = C + 2H_2_ and 2CO = C + CO_2_, respectively. The former is known to be a structure-sensitive reaction, being highly affected by the size of nickel crystallites.^58^ As for all the samples, the H_2_/CO molar ratio was lower than 1, and DMD seems to be limited. According to Dębek et al.,^56^ in the reaction of CH_4_/CO_2_/Ar = 2:1:7 with GHSV = 20,000 h^–1^, direct methane decomposition strongly influences DRM reaction at moderate temperatures, while at 700 °C the process is mostly affected by the simultaneous occurrence of the RWGS and Boudouard reactions.

#### 3.2.2 In Situ XAS–XRD during Excess-Methane Dry Reforming

**Figure 9:**
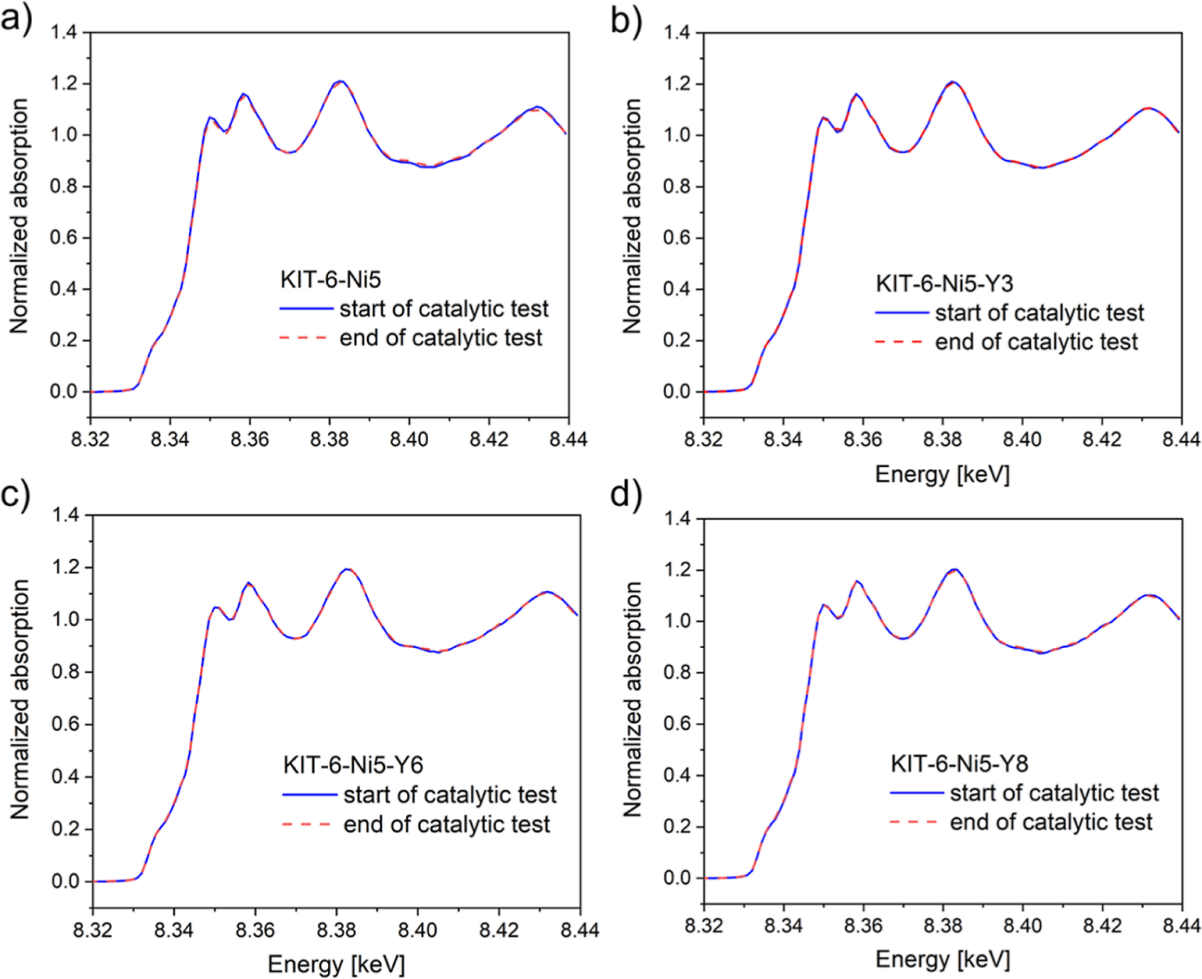
In situ Ni K-edge XANES from 8.32 to 8.44 keV was registered for the studied catalysts. The solid blue line represents the first spectra recorded when the excess-methane dry reforming started. The dashed red line refers to the last spectra recorded after 45 min of excess-methane dry reforming.

To identify possible oxidation changes and to understand the cause of catalyst deactivation, an in situ XAS–XRD analysis was carried out. For each sample, excess-methane dry reforming was monitored for 45 min. During this time, XRD revealed the formation of carbon by the successively growing intensity of the (002), (101), and (110) planes of graphite at 2θ = 5.7, 9.5, and 15.7°, respectively (Figure S13, Table S2, Supporting Information). The rate of carbon formation depends on the Ni crystallite size and is more difficult to initiate on smaller particles. The thermally sintered nickel particles (obtained during reduction at 750 °C for 90 min) were large enough to promote coke formation during CO_2_ reforming with excess of methane. Once the samples were subjected to the gas mixture of DRM, no significant increase in Ni crystallite size was observed after 45 min (Figure S14, Supporting Information). Large Ni particles, present in yttrium-promoted catalysts, limited the Ostwald ripening (chemical sintering) of Ni atoms, but in turn, they facilitated the formation of coke. The KIT-6-Ni5 catalyst had a certain resistance to both, i.e., extensive Ni particle growth and formation of the graphitic type of carbon. XANES analysis of the in situ monitored catalysts clearly showed that the Ni phase remained reduced (Figure 9a–d), which suggests that the rate of carbon encapsulation of the metallic particles was much faster than the rate of migration of Ni atoms and particle growth. Since the CH_4_ and CO_2_ conversions exhibited only a slight deactivation by time-on-stream, it appears that some metallic Ni particles are still accessible to play a role as the active phase in the catalytic reaction. To explain this phenomenon, one can follow the suggestion of Ruckenstein and Wang^59^ that the Rh/Y_2_O_3_ catalysts form a quasi-stationary concentration of metal centers as a result of controlling the rate of oxidation and reduction of metal particles, which would keep the metallic phase available due to the presence of a RhYO_3_ phase. Analogically, the formation of NiYO_3_ observed in the current study could be the reason for stable catalytic behavior as the strong Ni interactions with this compound can suppress metal sintering and moderate rates of reduction and oxidation during excess-methane dry reforming.

The spent samples showed only minor differences in the structural parameters obtained by EXAFS compared to the reduced state, showing a Ni–Ni coordination number close to 12 (Table S3, Supporting Information). This again confirms that the catalysts were resistant to oxidation under DRM reaction conditions. Moreover, no significant differences were observed between the EXAFS collected after excess-methane dry reforming (EXAFS3) and after the reaction when cooled down to 50 °C (EXAFS4) (Figure S1, Supporting Information). This implies that no significant thermal effects influenced the experimental data.

### 3.3 Characterization of the Spent Catalysts

**Figure 10:**
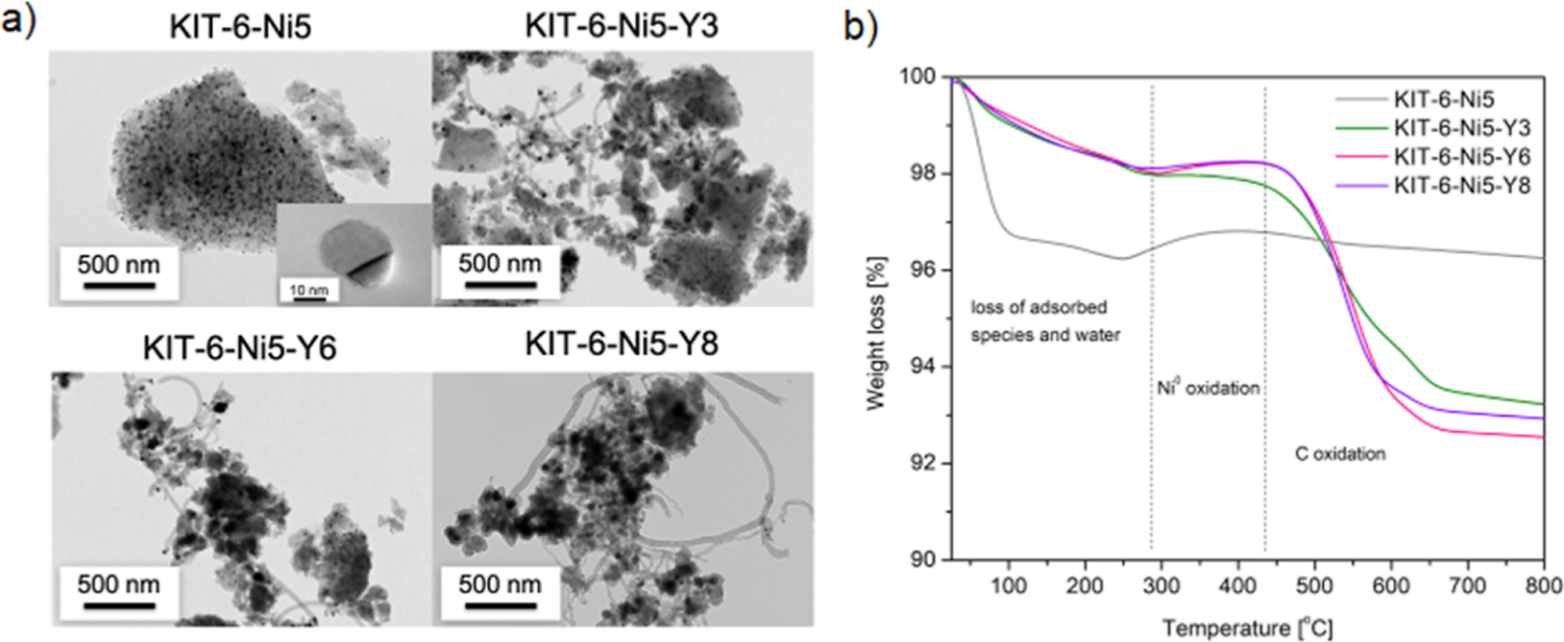
HRTEM micrographs and TGA curves of the spent catalysts (after 350 min of excess-methane dry reforming).

The formed carbon (caused by the occurrence of side reactions) is an important aspect in the discussion of the lifetime of the catalysts and on selectivity. When the surface of the catalyst has an affinity for carbon formation, the active phase (in this case metallic nickel) begins to be coated with coke and can be deactivated within a certain time. To analyze the type of carbonaceous species formed, the spent catalysts were examined by HRTEM (Figure 10a). Different types of carbon can be formed at dry reforming conditions, i.e., amorphous carbon, filamentous carbon, or graphitic carbon.^60,61^ The unpromoted catalyst showed less coke formation compared to yttrium-promoted samples since only a few Ni particles were encapsulated by carbon layers. The materials promoted with yttrium revealed the presence of a filamentous graphitic type of carbon, showing a tip growth mechanism involving Ni nanoparticles.

TGA was performed for the samples after testing in excess-methane dry reforming for 350 min. In Figure 10b, three main temperature zones can be distinguished: (i) loss of adsorbed species (H_2_O and/or CO_2_) below ca. 300 °C, (ii) Ni^0^ oxidation between 300 and 450 °C, and (iii) C-oxidation above ca. 450 °C.^62^ These regions are only approximate, as amorphous carbon can be oxidized already at temperatures lower than 300 °C in the presence of a metal catalyst.^63^ It is well known that the most deactivating type of coke is the one with the highest oxidation temperature. Therefore, the weight loss was calculated from the third zone (an individual temperature for each sample) to exclude interference from the loss of adsorbed species and Ni^0^ oxidation. KIT-6-Ni5 showed only 0.6% mass loss, and as confirmed by HRTEM, carbon filaments were hardly observed. The catalysts promoted with yttrium demonstrated a clear loss of weight, originating from the presence of filamentous carbon, such as nanotubes, and the following sequence of mass loss can be registered: 4.7% (KIT-6-Ni5-Y3) < 5.3% (KIT-6-Ni5-Y8) < 5.7% (KIT-6-Ni5-Y6). A clear correlation between the CH_4_ consumption rate and the amount of graphitic-like carbon formed can be observed. The relatively small Ni particles hindered the carbon-forming reaction, which involves the presence of methane, i.e., direct methane decomposition (CH_4_ = C + 2H_2_). Recalling the results from catalytic DRM, a relatively large amount of CH_4_ was not converted by the catalysts, and the production of H_2_ was lower than that of CO. Furthermore, the fact that the weight loss was less than 8%, retaining more than 92%, shows that the methane decomposition reaction had no major effect on the studied catalysts. Nevertheless, a clear correlation between increasing nickel crystallite size and the amount of produced carbon implies the occurrence of this reaction, but as mentioned above, to a limited extent.

## 4 Conclusions

In this work, a series of KIT-6-Ni5-Y*x* (*x* = 3, 6, 8) supported catalysts prepared by wet impregnation were used to demonstrate the effectiveness of promotion with yttrium. The performed experiments provide new insights into the role of yttrium in Ni-based catalysts during reduction in hydrogen and excess-methane dry reforming.

Various structural characterization methods and in-depth analysis via in situ Ni K-edge XAS–XRD revealed a stable size of nickel crystallites during DRM and formation of graphitic carbon. The analysis showed that promotion with yttrium affected the reduction rate of nickel oxide. The reduction rate has increased for bulk and weakly interacting with the support NiO, while a decrease in the rate was observed for NiO strongly interacting with KIT-6. Homogeneous distribution of nickel metal particles with the smallest average size did not necessarily lead to the highest activity and stability. It seems that the activity in excess-methane dry reforming was predominantly governed by the increase of basicity rather than the size of nickel crystallites or the number of active sites. The results showed that an increase in total basicity led to more stable performance in the studied reaction, and a direct correlation between the DF and the total number of basic sites was drawn. Strong interactions between nickel and yttrium compounds tend to increase the stability of the corresponding supported catalysts. The best-performing catalyst, i.e., KIT-6-Ni5-Y6, showed high activity and selectivity under excess-methane dry reforming, owing to the superior total number of basic sites. The obtained H_2_/CO ratio was stable and in the range of 0.83–0.7, which can be appropriate for synthesis gas used in Fischer–Tropsch synthesis over a Fe-based catalyst.

This work offers a new choice of yttrium-promoted Ni-based mesoporous silica catalysts. Synthesis gas is one of the most important intermediates to produce chemicals and fuels. Excess-methane dry reforming was studied over KIT-6-Ni-Y catalysts. Such catalysts may offer a future alternative for upgrading low-quality natural gas or biogas to syngas.

## Supporting Information Available

Experimental part presenting the calculations of conversions, the rates of reaction, STY, and the number of active sites; experimental procedure of XAS–XRD experiments; flowsheet of the setup used for XAS–XRD experiments; Ni K-edge XANES spectra collected for KIT-6-Ni5, KIT-6-Ni5-Y3, KIT-5-Ni5-Y6, and KIT-5-Ni5-Y8 during reduction from 50 °C (NiO phase) to 750 °C (Ni^0^ phase); XANES LCF fraction against temperature for estimation of the rate of reduction; H_2_-TPR profiles of the studied catalysts; Ni K-edge Fourier-transformed *k*^3^-weighted EXAFS spectra of two standards, i.e., NiO and Ni foil; and illustration of the synthesis procedure of yttrium-promoted Ni-based KIT-6 catalysts (PDF)
