# Supplementary material for: Excess-Methane CO2 Reforming over Reduced KIT-6-Ni-Y Mesoporous Silicas Monitored by In Situ XAS–XRD
Source: Energy Fuels. 2023 Nov 17;37(23):18952–67. doi: 10.1021/acs.energyfuels.3c02994 (PMC10714349; doi:10.1021/acs.energyfuels.3c02994)
Supplement: Supplementary file 1 — ef3c02994_si_001.pdf [file ef3c02994_si_001.pdf]

Supporting Information

Excess-methane CO_2 reforming over reduced KIT-6-Ni-Y mesoporous silicas monitored by *in-situ* XAS-XRD

Katarzyna Świrk Da Costa [a*], *Paulina Summa* [b,c,1], *Jithin Gopakumar* [a], *Youri van Valen* [a], *Patrick Da Costa* [b], *Magnus Rønning* [a*]

[a] Norwegian University of Science and Technology (NTNU), Department of Chemical Engineering, 7491 Trondheim, Norway

[b] Sorbonne Univeristé, Institut Jean Le Rond d'Alembert, CNRS UMR 7190, 78210 Saint-Cyr-l'Ecole, France

[c] AGH University of Science and Technology, Faculty of Energy and Fuels, 30-059 Cracow, Poland

* e-mail: katarzyna.swirk@ntnu.no, magnus.ronning@ntnu.no

[1] Present address: Fritz-Haber-Institut der Max-Planck-Gesellschaft, Faradayweg 4-6, 14195 Berlin, Germany

Table of Contents

Supplementary text, figures and tables *3*

1. Experimental part 3

1. *Ex-situ* and *In-situ* characterization results 6

Supplementary text, figures and tables

1. Experimental part

In order to acquire CH_4, CO_2, conversions, H_2/CO molar ratio, Site Time Yield, CH_4, CO_2 consumption rate the following Eqs. (1-7) were employed:

CH_4 conversion

$$X_{CH_4} = \left[\frac{F_{inlet} \cdot n_{CH_4^{in}} - F_{outlet} \cdot n_{CH_4^{out}}}{F_{inlet} \cdot n_{CH_4^{in}}}\right] \qquad \text{(Eq.1)}$$

CO_2 conversion

$$X_{CO_2} = \left[\frac{F_{inlet} \cdot n_{CO_2^{in}} - F_{outlet} \cdot n_{CO_2^{out}}}{F_{inlet} \cdot n_{CO_2^{in}}}\right] \qquad \text{(Eq.2)}$$

H_2/CO molar ratio

$$\frac{H_2}{CO} = \left[\frac{n_{H_2^{out}}}{n_{CO_2^{out}}}\right] \qquad \text{(Eq.3)}$$

Where:

$n_{CH4/CO2}^{in/out}$ are the respective mole fractions of CH_4 and CO_2 in the inlet and outlet, F_{inlet} and F_{outlet} represents the total gas flow rate [mL min^{-1}], respectively. Similarly, for H_2 and CO.

$$STY_{H_2} = 2 \cdot \frac{F^0 \cdot C_{CH_4}^0 \cdot X_{in-CH_4}}{22.414 \cdot N_{Ni}} \qquad \text{(Eq.4)}$$

$$N_{Ni} = \frac{g \cdot W_{Ni}}{M_{Ni}} \cdot D_{Ni} \qquad \text{(Eq.5)}$$

Where:

F^0 is the inlet flow of reactants [L s^{-1}],

c^0_{CH4} is the inlet concentration of methane in the reactant's mixture

$Xi_{in\text{-}CH4}$ is the initial CH_4 conversion at 700°C

22.414 is the volume of one mole of gas at standard conditions [L mol^{-1}]

N_{ni} is the number of moles of Ni active sites

g is the mass of catalyst,

W_{Ni} is the weight fraction of Ni in the sample as determined by ICP-OES,

M_{Ni} is the molar mass of Ni,

D_{Ni} is the Ni^0 dispersion

CH_4 consumption rate

$$r_{CH4} = \frac{WHSV \cdot f_{CH_4} \cdot X_{CH_4}}{3600 \cdot 22414} \cdot 1000 \qquad \text{(Eq.6)}$$

CO_2 consumption rate

$$r_{CH4} = \frac{WHSV \cdot f_{CO_2} \cdot X_{CO_2}}{3600 \cdot 22414} \cdot 1000 \qquad \text{(Eq.7)}$$

Where:

$WHSV$ is the weight hourly space velocity [mL g^{-1} h^{-1}],

f_{CH_4/CO_2} is the fraction of methane/carbon dioxide in the reactant mixture

X_{CH4} is CH_4 conversion at 700°C

X_{CO2} is CO_2 conversion at 700°C

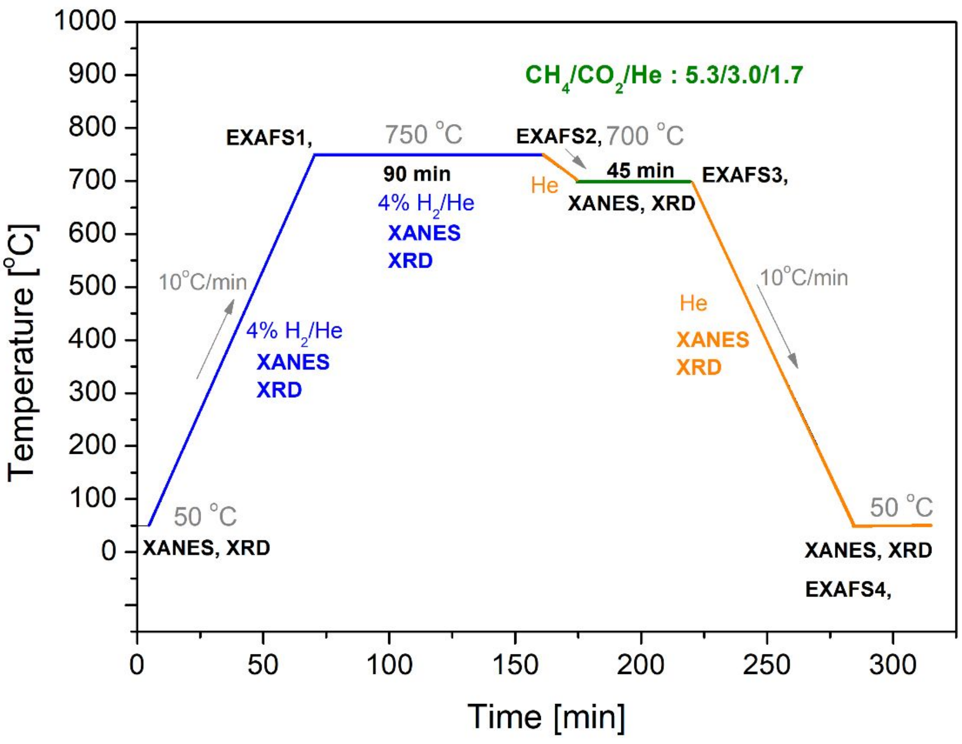

Fig. S1 Experimental procedure containing the measurement steps performed during *in-situ* XAS-XRD experiments.

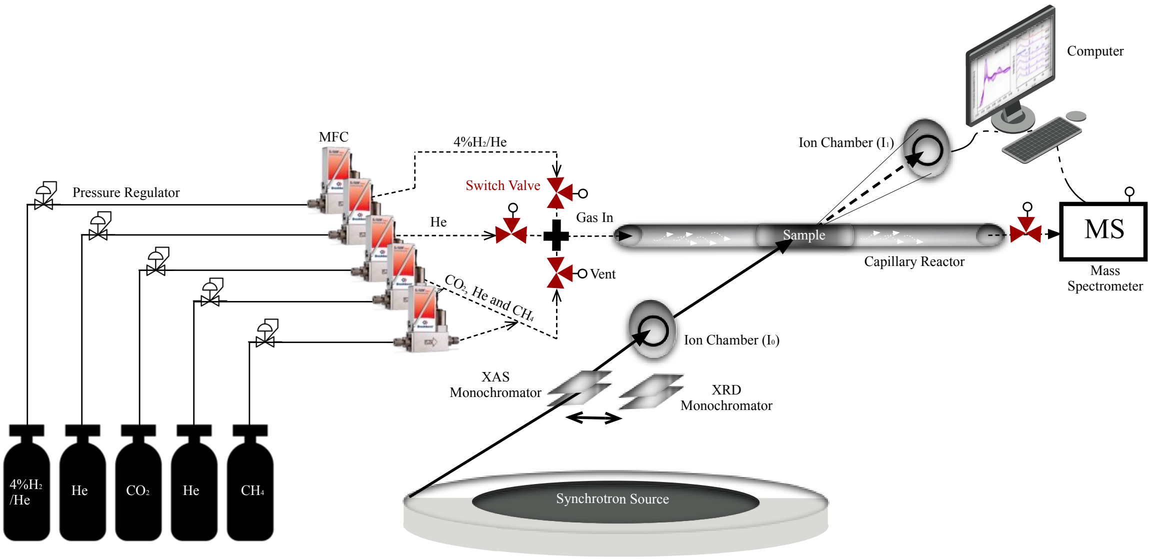

Fig. S2 Flowsheet of the experimental setup used for combined XAS-XRD at BM31.

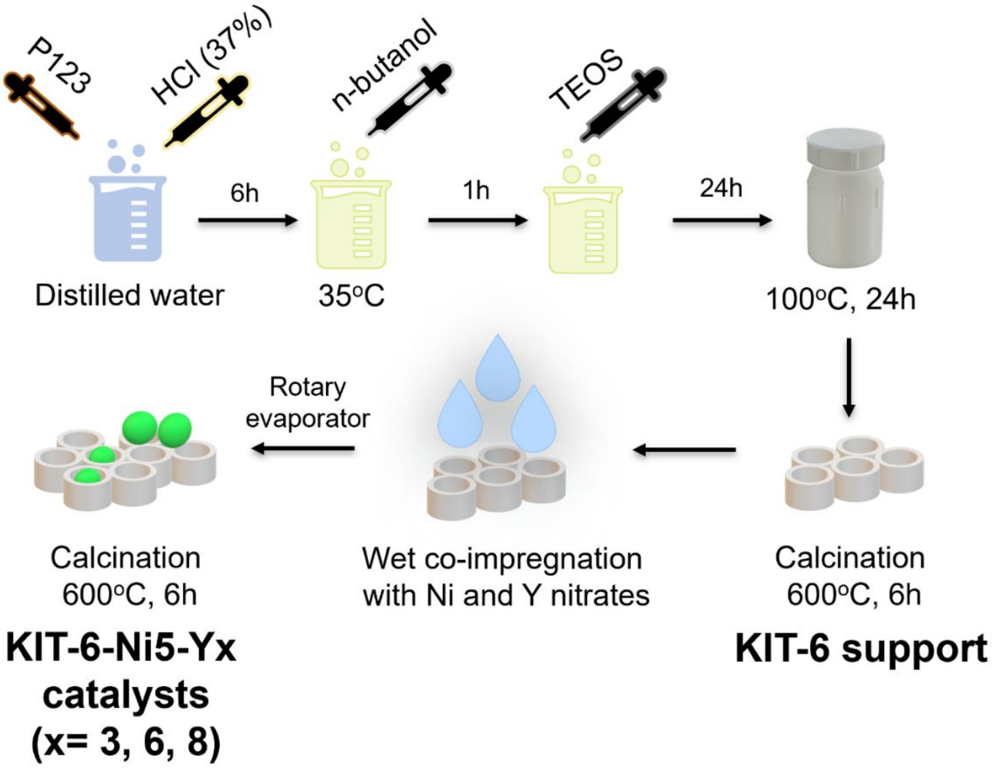

Scheme S1. Schematic illustration of synthesis steps of yttrium-promoted Ni-based KIT-6 catalysts. P123 refers to Pluronic P123, i.e., the symmetric triblock copolymer which constitutes of poly(ethylene oxide)(PEO) and poly (propylene oxide) (PPO). TEOS stands for tetraethyl orthosilicate. The produced catalysts are named as: KIT-6-Ni5-Yx, where x represents the loading of yttrium.

1. *Ex-situ* and *in-situ* characterization results

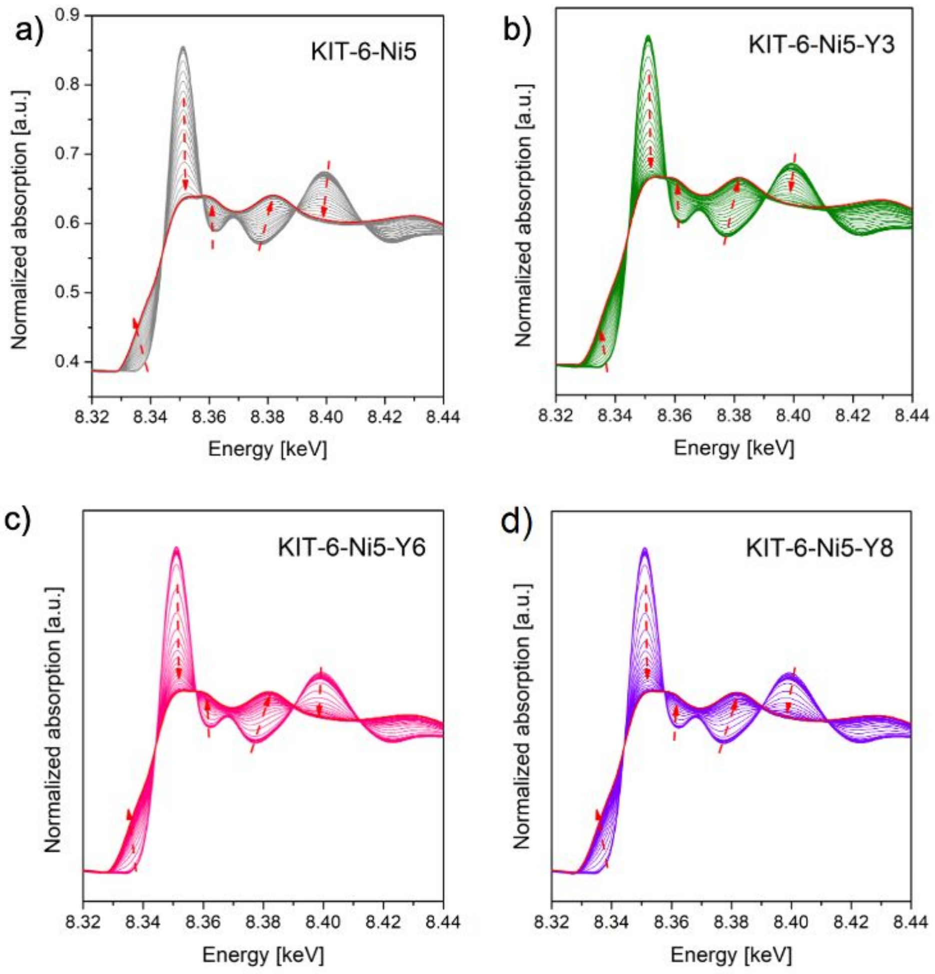

Fig. S3 Ni K-edge XANES spectra collected for KIT-6-Ni5, KIT-6-Ni5-Y3, KIT-5-Ni5-Y6 and KIT-5-Ni5-Y8 during reduction from 50°C (NiO phase) to 750°C (Ni^0 phase).

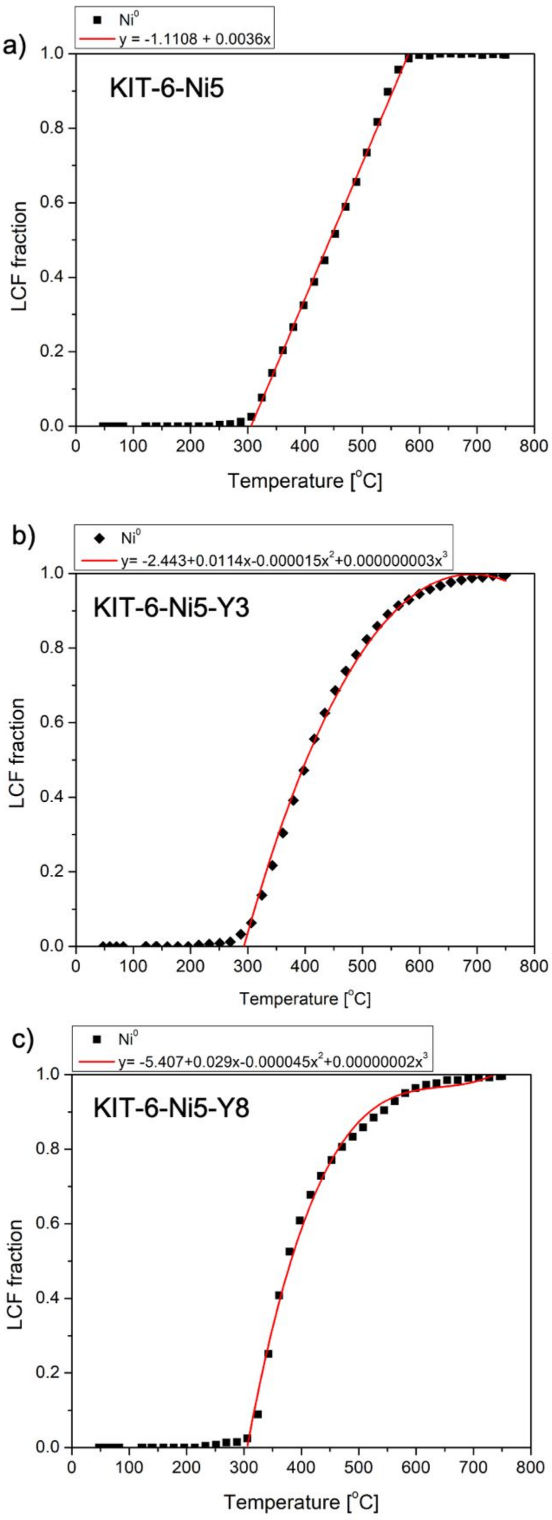

Fig. S4 XANES LCF fraction against temperature for estimation of the rate of reduction.

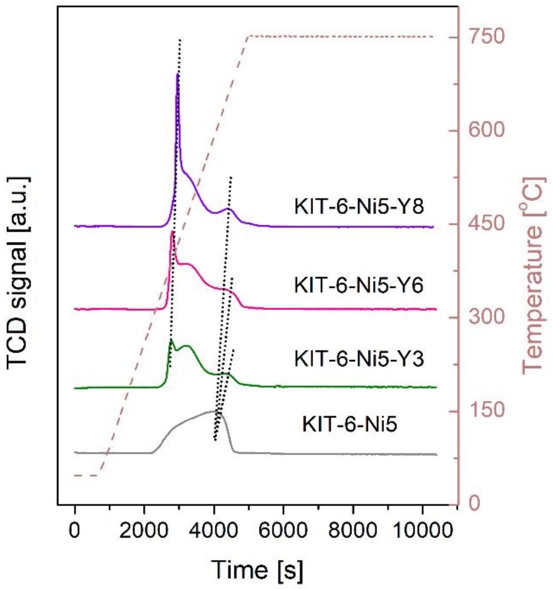

Fig. S5 TPR-H_2 profiles for KIT-6-Ni5, KIT-6-Ni5-Y3, KIT-5-Ni5-Y6 and KIT-5-Ni5-Y8.

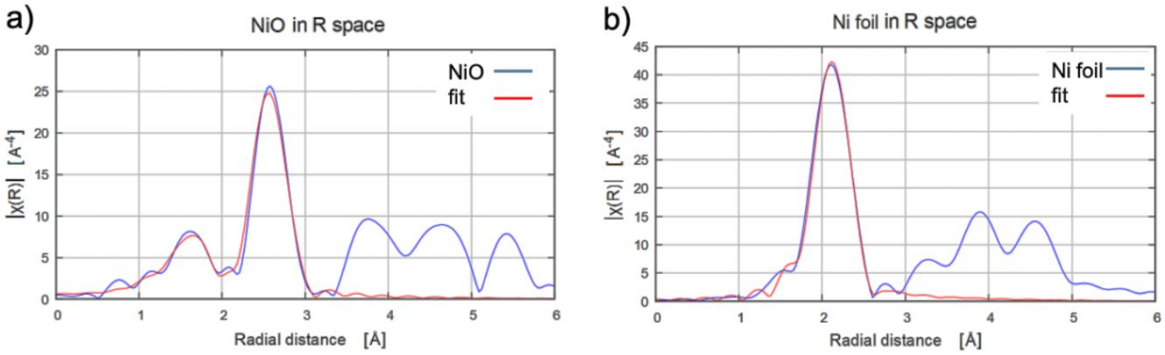

Fig. S6 Ni K-edge Fourier transformed k^3-weighted EXAFS spectra of standards: (a) NiO, (b) Ni foil.

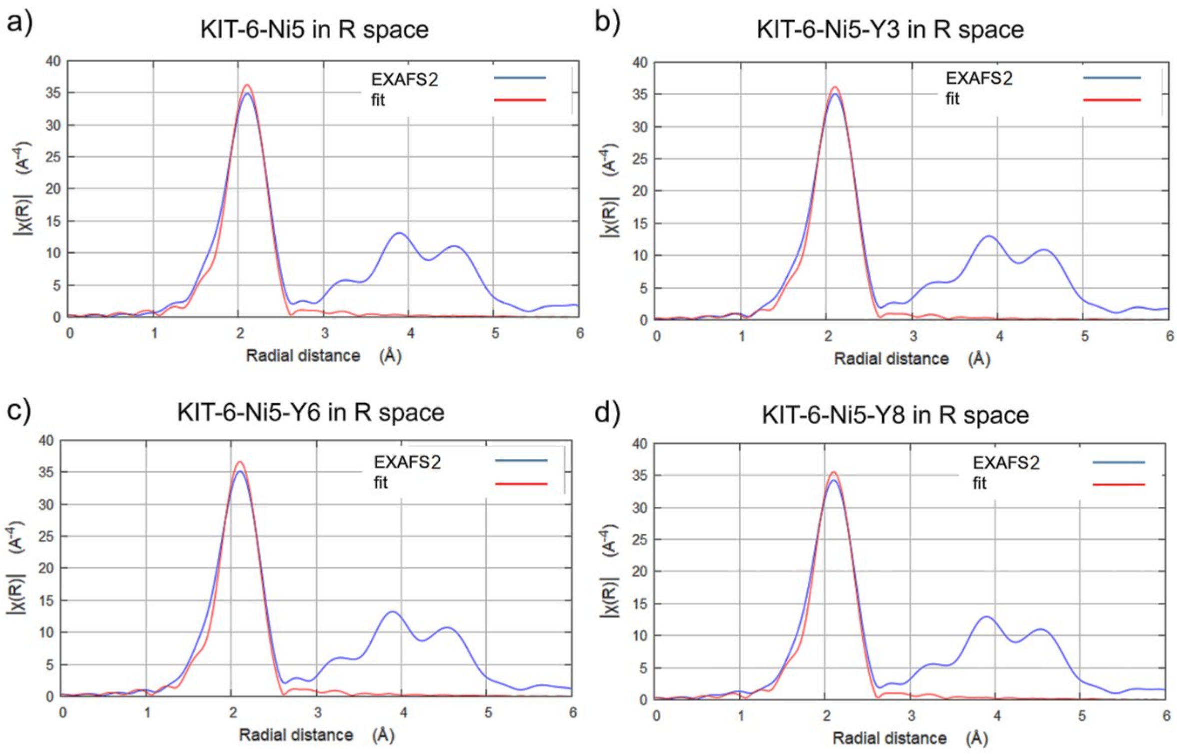

Fig. S7 Ni K-edge Fourier transformed k^3-weighted EXAFS spectra in R space and the best fitting results for *in-situ* reduced catalysts (EXAFS2 see **Fig. S1**): (a) KIT-6-Ni5, (b) KIT-6-Ni5-Y3, (c) KIT-6-Ni5-Y6, (d) KIT-6-Ni5-Y8.

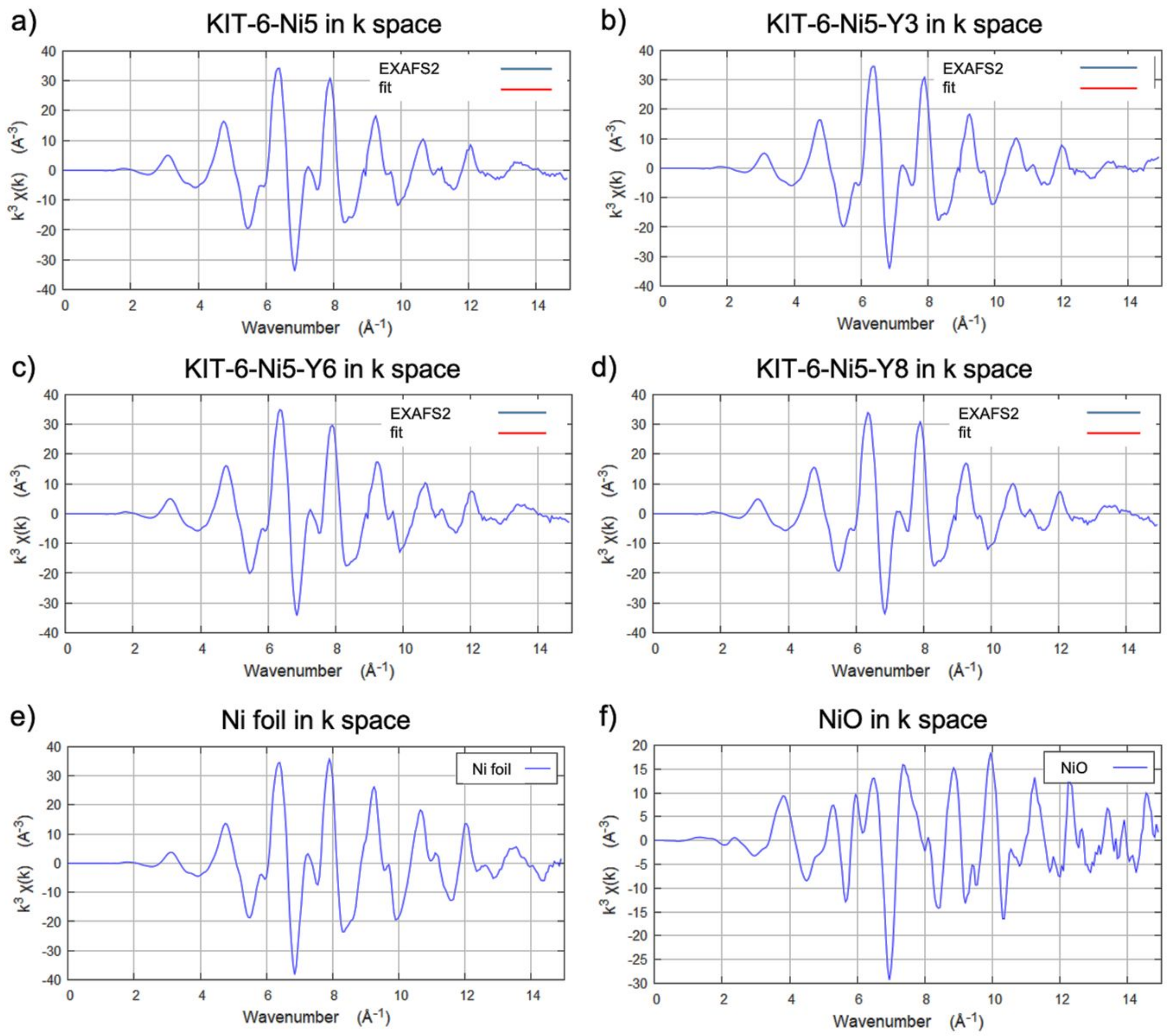

Fig. S8 Ni K-edge Fourier transformed k^3-weighted EXAFS spectra in k space for *in-situ* reduced catalysts: (a) KIT-6-Ni5, (b) KIT-6-Ni5-Y3, (c) KIT-6-Ni5-Y6, (d) KIT-6-Ni5-Y8; and the standards collected at room temperature: (e) Ni foil, and (f) NiO.

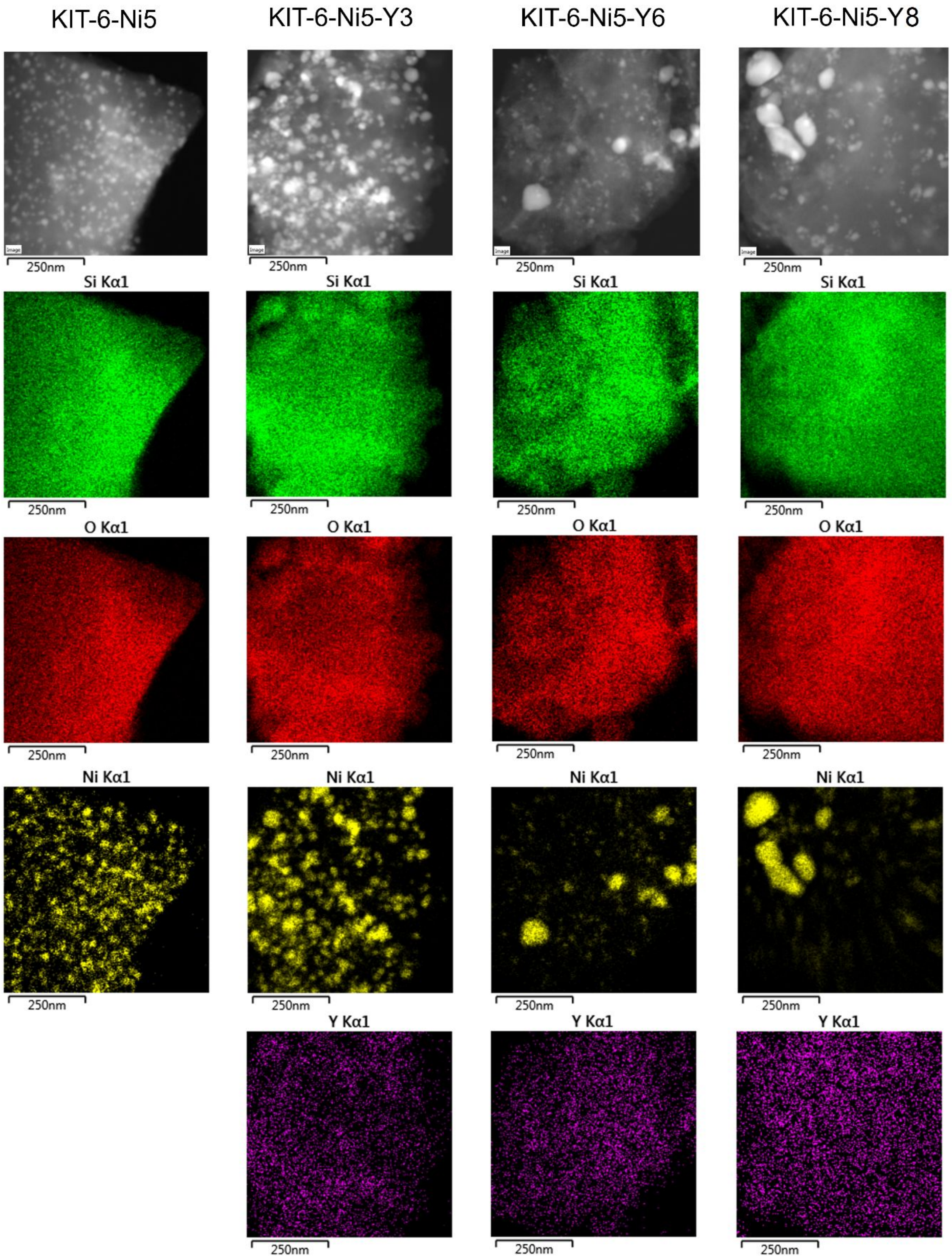

Fig. S9 EDS elemental mapping of the studied catalysts.

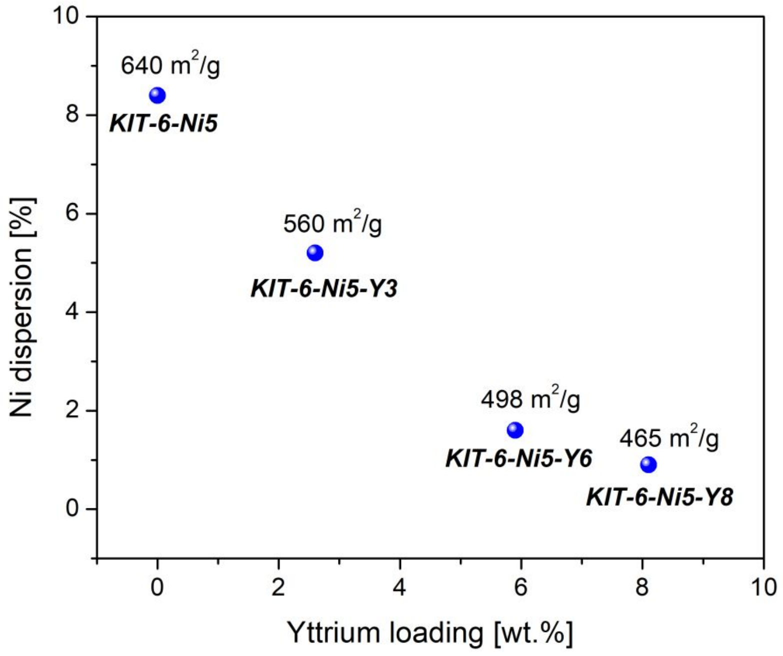

Fig. S10 Correlation between yttrium loading and Ni dispersion.

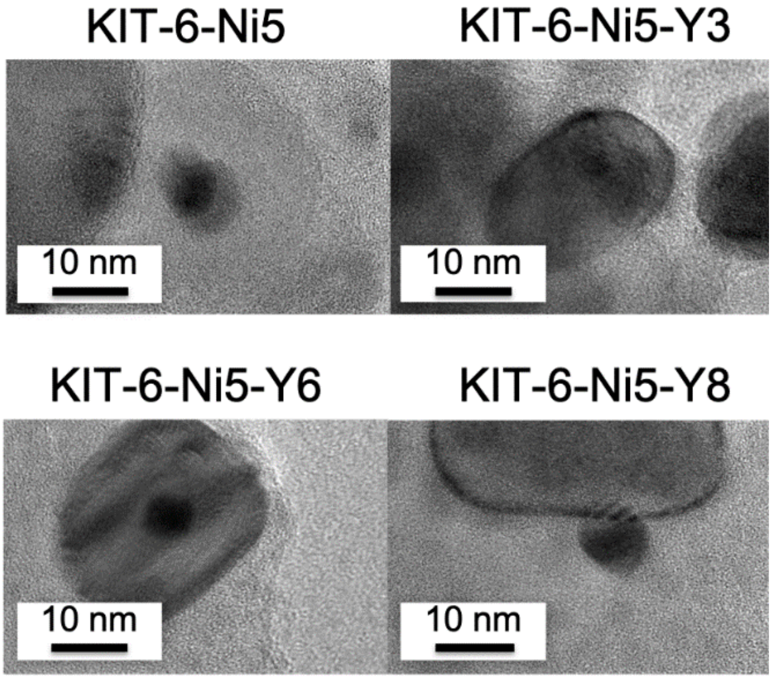

Fig. S11 Examples of agglomerates of nickel particles in TEM images.

Table S1. Basicity of different yttrium promoted catalysts studied by TPD-CO_2 technique.

**Catalyst (loading of Ni, Y)**	**Reaction**	**Basicity**<br><br>**Peak position, Basic sites: Weak (W), Medium (M), Strong (S)**<br><br>**Total (T)**	**Influence of yttrium on basicity**	**Reference**
**NiO–ZrOm–Y (Ni: 10 wt.%[b], Y:10 wt.%[a])**	Dry reforming of methane	W: 150°C (36.9%)<br><br>M: 240°C (55.4%),<br><br>S: 380°C (13%)<br><br>T: 100 $\mu mol_{CO2}/g_{cat}$	Formation of new weak basic sites compared to Y-free catalyst. Yttrium containing catalyst showed higher total basicity	Wang et al. [1]
**HTNi-Y1.5 (Ni: 14 wt.%[b], Y:1.5 wt.% [b])**	Dry reforming of methane	W: 135°C (14.5 %)<br><br>M: 203°C (47.1 %),<br><br>S: 316°C (38.4 %)<br><br>T: 77.1 $\mu mol_{CO2}/g_{cat}$	Increased fraction of medium basic sites. This catalyst also showed smaller Ni crystallite size and higher specific surface area compared to other tested samples	Świrk et al.[2]
**Ni8YSZ (Ni: 5.6 wt.% [b], Y:4.3 wt.% [b])**	Aqueous-phase reforming of methanol	W: 90-180°C (18.1 $\mu mol_{CO2}/g_{cat}$),<br><br>M: 180-400°C (7.9 $\mu mol_{CO2}/g_{cat}$),<br><br>S: -<br><br>T: 26 $\mu mol_{CO2}/g_{cat}$	When Y is used the intermediate basicity increased in respect to the bare sample and the competitive Ca-promoted sample. Total basicity is similar to Ca-promoted sample	Goma et al.[3]
**Ni/Al-15Y (Ni:13.4 wt.% [b], Y: 14 wt.% [b])**	CO_2 hydrogenation to methane	W: -<br><br>M: 242°C, 0.091 $mmol_{CO2}/g_{cat}$<br><br>S: 344°C, 0.056 $mmol_{CO2}/g_{cat}$<br><br>T:0.147 $mmol_{CO2}/g_{cat}$	High CO_2 desorption on medium basic sites. Total basicity comparable to other tested samples	Battumur et al. [4]
**10Ni/10Y/SBA-16 (Ni: 10 wt.% [a], Y: 15 wt.% [a])**	CO_2 hydrogenation to methane	W: 95°C (4.3 $\mu mol_{CO2}/g_{cat}$ = 6.2%)<br><br>M: 240°C (65.5 $\mu mol_{CO2}/g_{cat}$= 93.8%)<br><br>S: -<br><br>T: 69.8 $\mu mol_{CO2}/g_{cat}$	An increase of medium basic sites with a corresponding decrease of weak basic sites	Sun et al.[5]

[a] Nominal loading
[b] Actual loading

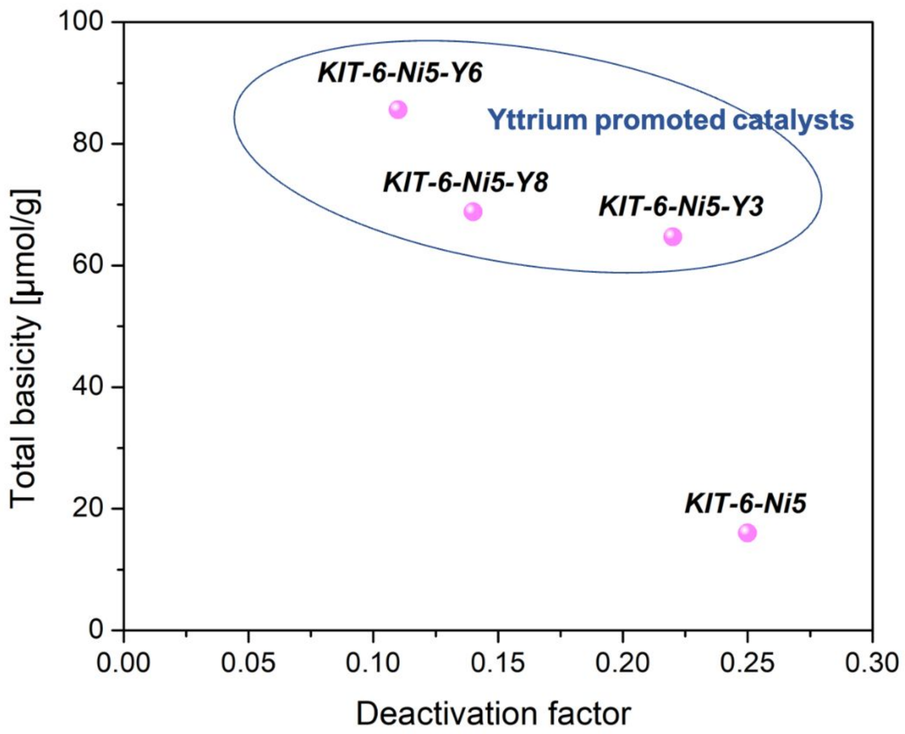

Fig. S12 Deactivation factor correlated with total basicity of the reduced materials.

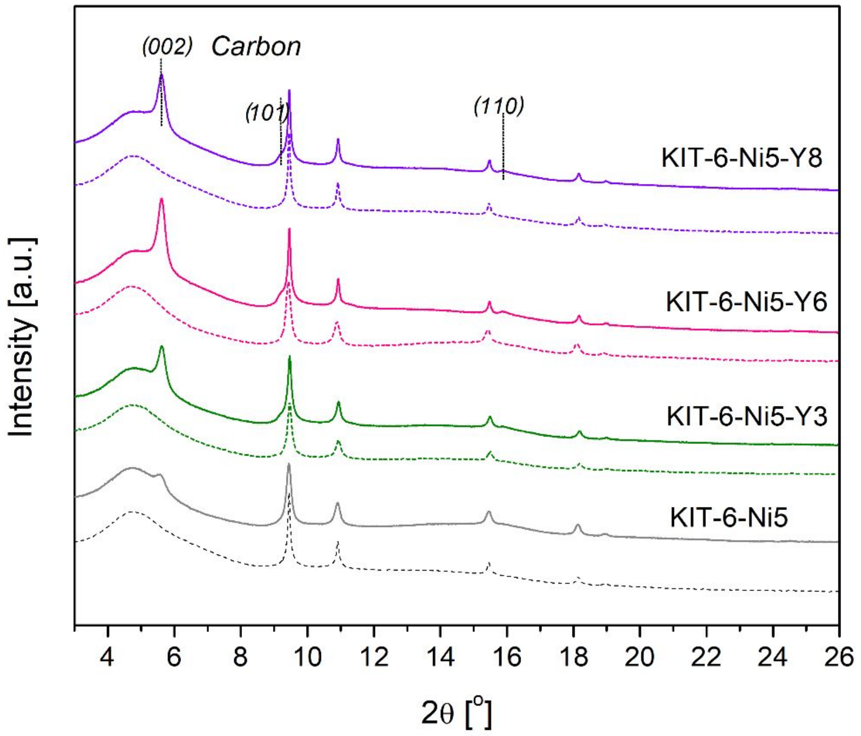

Fig. S13 Powder X-ray diffractograms collected using synchrotron radiation (λ = 0.0338 nm). Dashed line represents beginning of the catalytic reaction, a solid line refers to the end of catalytic process.

Table S2 X-ray diffraction data for the detected phases at λ = 0.0338 nm and λ = 0.15406 nm.

**Cubic nickel oxide**	**(hkl)**	**d-spacing [Å]**	**2θ [deg] λ = 0.0338 [nm]**	**2θ [deg] λ = 0.15406[nm]**
**1**	111	2.41	8.07	37.23
**2**	200	2.09	9.28	43.25
**3**	220	1.47	13.14	62.83
**4**	311	1.26	15.42	75.35
**5**	222	1.21	16.11	79.34

**Cubic metallic nickel**	**(hkl)**	**d-spacing [Å]**	**2θ [deg] λ = 0.0338 [nm]**	**2θ [deg] λ = 0.15406[nm]**
**1**	111	2.04	9.53	44.49
**2**	200	1.76	11.01	51.85
**3**	220	1.25	15.59	76.38

**Graphite**	**(hkl)**	**d-spacing [Å]**	**2θ [deg] λ = 0.0338 [nm]**	**2θ [deg] λ = 0.15406[nm]**
**1**	002	3.39	5.70	26.23
**2**	101	2.04	9.50	44.37
**5**	110	1.24	15.73	77.18

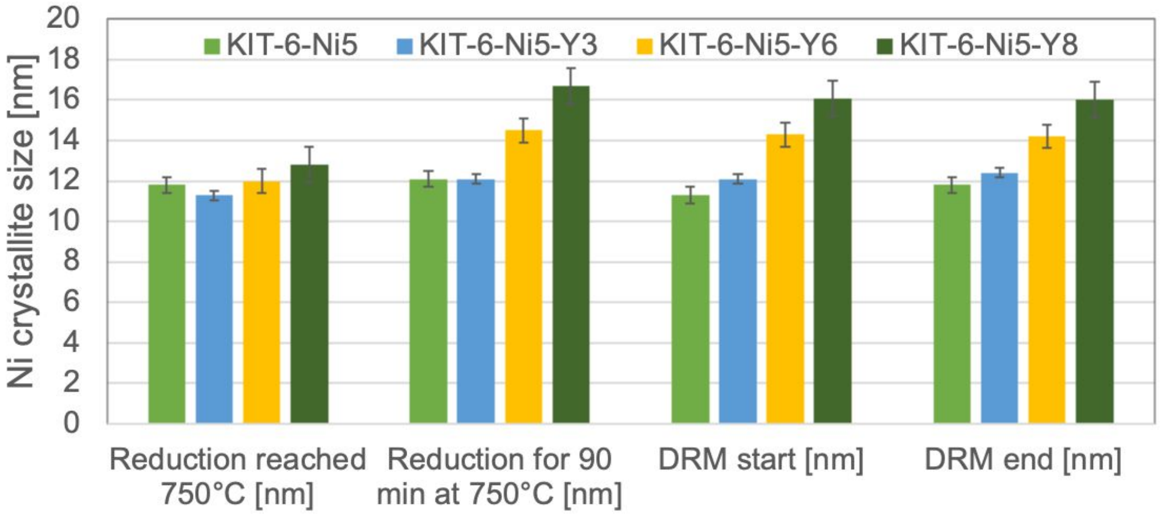

Fig. S14 Nickel crystallite size estimated by the Scherrer equation from the collected XRD diffractograms. The error bars represent experimental error obtained during the calculations. One can see a remarkable Ni particle growth for KIT-6-Ni5-Y6 and KIT-6-Ni5-Y8 after 90 min of reduction at 750°C.

Table S3 Structural parameters obtained by EXAFS fitting (Ni K-edge) for spent KIT-6-Ni5-Yx catalysts.

**Sample**	**State**	**Bond**	**Coordination number**	**R [Å]**	σ^2 **[Å^2]**	***R*-factor**
**KIT-6-Ni5**	Spent	Ni-Ni	12.0 ±0.1	2.5 ±0.01	0.008 ±0.001	0.030
**KIT-6-Ni5-Y3**	Spent	Ni-Ni	12.0±0.1	2.5±0.01	0.008±0.001	0.031
**KIT-6-Ni5-Y8**	Spent	Ni-Ni	12.0 ±0.1	2.5±0.01	0.008 ±0.001	0.028

References

(1) Wang, Y.; Li, L.; Wang, Y.; Da Costa, P.; Hu, C. Highly Carbon-Resistant Y Doped NiO–ZrOm Catalysts for Dry Reforming of Methane. *Catalysts* **2019**, *9* (12), 1–13. https://doi.org/10.3390/catal9121055.

(2) Świrk, K.; Gálvez, M. E.; Motak, M.; Grzybek, T.; Rønning, M.; Da Costa, P.; Costa, P. Da. Yttrium Promoted Ni-Based Double-Layered Hydroxides for Dry Methane Reforming. *J. CO2 Util.* **2018**, *27* (August), 247–258. https://doi.org/10.1016/j.jcou.2018.08.004.

(3) Goma, D.; Delgado, J. J.; Lefferts, L.; Faria, J.; Calvino, J. J.; Cauqui, M. Á. Catalytic Performance of Ni/CeO_2/X-ZrO_2 (X = Ca, Y) Catalysts in the Aqueous-Phase Reforming of Methanol. *Nanomaterials* **2019**, *9* (11), 1582–1599. https://doi.org/10.3390/nano9111582.

(4) Battumur, N.; Sergelenbaatar, N.; Bold, T.; Byambajav, E. Cerium-Promoted Nickel Catalysts Supported on Yttrium-Doped γ-Alumina for Carbon Dioxide Methanation. *J. CO2 Util.* **2023**, *68* (December 2022), 102380–102387. https://doi.org/10.1016/j.jcou.2022.102380.

(5) Sun, C.; Świrk, K.; Wang, Y.; Li, L.; Fabbiani, M.; Hulea, V.; Rønning, M.; Hu, C.; Da Costa, P. Unraveling Catalytic Properties by Yttrium Promotion on Mesoporous SBA-16 Supported Nickel Catalysts towards CO_2 Methanation. *Fuel* **2022**, *317* (October 2021), 122829–122842. https://doi.org/10.1016/j.fuel.2021.122829.
